# Microbial diversity and biosignatures of amorphous silica deposits in orthoquartzite caves

**DOI:** 10.1038/s41598-018-35532-y

**Published:** 2018-12-04

**Authors:** Francesco Sauro, Martina Cappelletti, Daniele Ghezzi, Andrea Columbu, Pei-Ying Hong, Hosam Mamoon Zowawi, Cristina Carbone, Leonardo Piccini, Freddy Vergara, Davide Zannoni, Jo De Waele

**Affiliations:** 1 Department of Biological Geological and Environmental Sciences, University of Bologna, 40126 Bologna, Italy; 2 La Venta Geographic Explorations Association, 31100 Treviso, Italy; 3 Department of Pharmacy and BioTechnology, University of Bologna, Bologna, 40126 Italy; 4 Division of Biological and Environmental Science and Engineering, King Abdullah University of Science and Technology (KAUST), Thuwal, 23955-6900 Saudi Arabia; 5 The University of Queensland, Centre for Clinical Research (UQCCR), Herston, 4029 Australia; 6 College of Medicine, King Saud bin Abdulaziz University for Health Sciences, 3130, Riyadh, Saudi Arabia; 7 Department of Earth, Environment and Life, University of Genoa, Genoa, 16132 Italy; 8 Department of Earth Sciences, University of Florence, 50121 Florence, Italy; 9 Teraphosa Exploring Team, Puerto Ordaz, Venezuela

## Abstract

Chemical mobility of crystalline and amorphous SiO_2_ plays a fundamental role in several geochemical and biological processes, with silicate minerals being the most abundant components of the Earth’s crust. Although the oldest evidences of life on Earth are fossilized in microcrystalline silica deposits, little is known about the functional role that bacteria can exert on silica mobility at non-thermal and neutral pH conditions. Here, a microbial influence on silica mobilization event occurring in the Earth’s largest orthoquartzite cave is described. Transition from the pristine orthoquartzite to amorphous silica opaline precipitates in the form of stromatolite-like structures is documented through mineralogical, microscopic and geochemical analyses showing an increase of metals and other bioessential elements accompanied by permineralized bacterial cells and ultrastructures. Illumina sequencing of the 16S rRNA gene describes the bacterial diversity characterizing the consecutive amorphization steps to provide clues on the biogeochemical factors playing a role in the silica solubilization and precipitation processes. These results show that both quartz weathering and silica mobility are affected by chemotrophic bacterial communities, providing insights for the understanding of the silica cycle in the subsurface.

## Introduction

In the last two decades, understanding the functional role of microorganisms in quartz and silicate weathering and in the formation of biomediated amorphous silica deposits has emerged at the forefront of scientific investigation for gathering insights on the global silica cycle^1^. While silica precipitation processes have been extensively studied in hot spring systems where rapid cooling phenomena, steam loss and evaporation, mixing and pH changes in solutions cause the precipitation of amorphous silica in the form of hard siliceous sinters^2,3^, little is known about the biologically-mediated weathering affecting quartz-rich lithologies^4^ and the formation of silica stromatolites in non-thermal conditions^5–7^. Of particular interest are the microbial processes that are thought to have a direct role on the silica cycle in soils and in subsurface environments presenting stable physical-geochemical conditions with ambient temperature and neutral pH. Concurrently, the ability of microorganisms to enhance silica mobilization and to be entombed by amorphous silica are crucial for the comprehension of silica-microbe interactions in ancient natural environments, such as for some Precambrian microfossils^8^.

**Figure 1:**
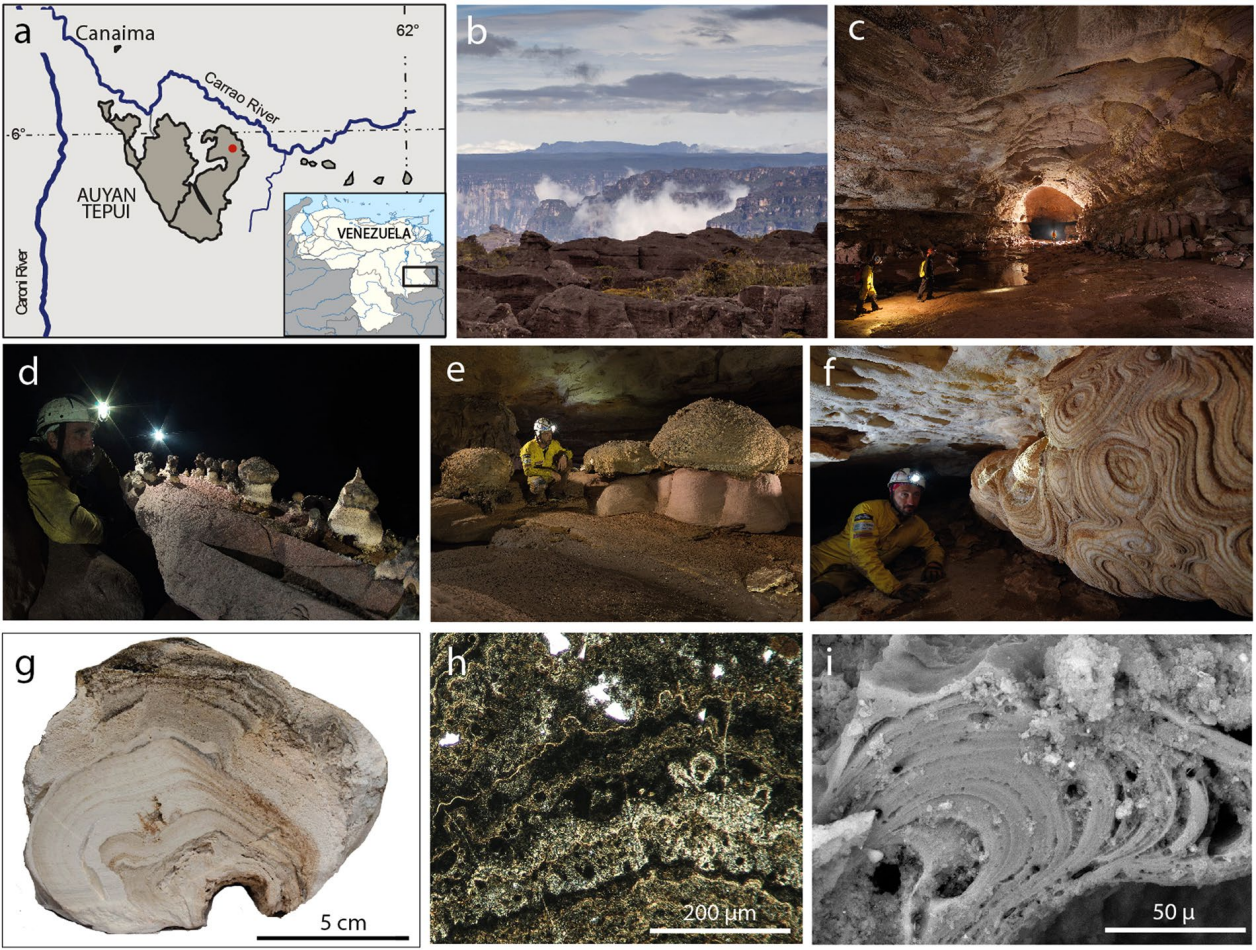
Study area, cave system and silica deposits. Located in the southeastern corner of Venezuela (**a**), the Auyan Tepui table mountain (**b**) hosts the 23 km-long Imawarì Yeuta cave system (**c** and red point in **a**). Examples of biologically mediated opaline silica deposits in Imawarì Yeuta cave: mushroom-like speleothems built by layered soft and highly porous amorphous silica (thinner and clearer at the base, blackish and wider on top) in the hydrologically inactive areas of the cave (**d**); massive silica stromatolite-like columnar formations growing on pinkish orthoquartzite boulders (**e**); giant deposits of opaline silica with concentric growth bands completely covering the orthoquartzite walls of the cave (**f**). In cross-section most of the deposits are characterized by layered porous opaline silica (**g**) with typical wavy and crinkled lamina and thin opaque lamina under plane polarized light (**h**) and single micro-columnar features visible with SEM (**i**). Photos are provided by La Venta Archive (**b**, N. Russo; **c**, R. Shone; **d** and **f**, V. Crobu; **e**, R. De Luca).

Subsurface caves in quartz-rich lithologies (orthoquartzites, metaquartzites, and granites) are characterized by enduring (i.e. thousands or millions of years), highly stable temperature and geochemical settings^9,10^; in this respect, detailed studies of these environments allow to understand the mechanisms through which microorganisms play a role in quartz dissolution and silica re-precipitation in colloidal forms. In 2013 the discovery of giant cave systems (Fig. 1, Supplementary Fig. S1) carved in the Precambrian^11^ orthoquartzitic table mountains (Gran Sabana, Venezuela), locally named *tepui*, allowed access to unique amorphous silica deposits with the extraordinary characteristic of growing in a geochemically stable, non-thermal, aphotic environment^6,7^ at approximately one hundred meters of depth below the *tepui* plateau surface. The ∼20–30 Ma old Imawarì Yeuta cave, discovered in the Auyan Tepui (Fig. 1a–c), is among the less accessible and most pristine places on Earth representing the longest^12,13^, and probably oldest, known cave system in quartz-rich lithologies. A heated scientific debate on the genesis of the cave has arisen^14,15^, since the extremely low solubility and dissolution rates of quartz would not allow the formation of such giant underground voids in geological times as in well-known carbonate karst terrains. The most common process of speleogenesis^13^ considers an extremely slow chemical weathering of the quartz intergranular boundaries, turning the orthoquartzite in a low cohesive and easily erodible material (arenization), after which piping and erosion can carve the subterranean conduits. Accordingly, the presence of important amounts of amorphous silica deposits with stromatolitic features have suggested that quartz dissolution versus amorphous silica precipitation is one of the main factors controlling the subsurface weathering of the orthoquartzite^12^. Under the stable physical-geochemical condition (at constant T of 15–18 °C and water pH of 5–6) characterizing *tepui* caves, quartz is characterized by both extremely low solubility and reaction kinetics^16^. Therefore, other processes, different from those occurring in hot springs, are required to explain the mobilization and re-precipitation of important amounts of SiO_2_ responsible for the formation of silica speleothems observed in Imawarì Yeuta cave^7^. All these deposits are composed of almost pure amorphous silica, currently developing on the weathered orthoquartzite walls and floors of the cave^17^. Before the discovery of the Imawarì Yeuta cave, amorphous silica speleothems were reported only in a few other caves of the Venezuelan *tepui*^6,7^, lava tubes^5,18^ and granite caves^9^ in other locations, but never in such amount and diversity (Fig. 1d–f).

Here, novel mineralogical (XRD), geochemical (XRF), morphological (SEM-FESEM), and microbiological (16S rRNA gene targeting NGS) analysis on silica samples from Imawarì Yeuta caves, are reported. The identification of complex microbial community structures together with morphological and geochemical biosignatures in the *tepui* caves reveals important clues on their involvement in quartz weathering and on the functional microbial mechanisms and biomineralization processes occurring in these extreme oligotrophic environments under constant physical-chemical conditions.

## Results

### Sampling environments and geochemistry

**Figure 2:**
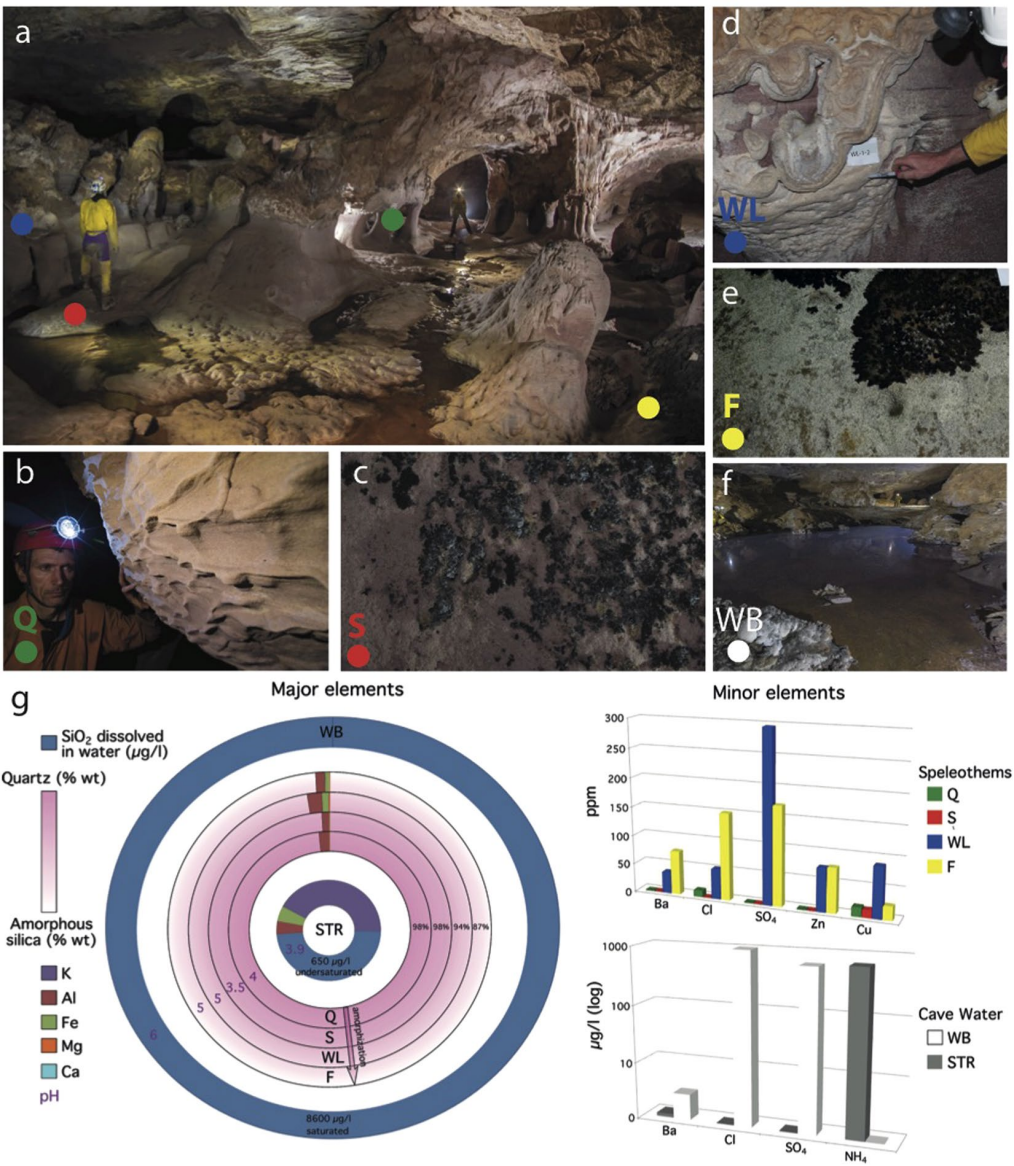
Sampling environments and geochemical characteristics. Colored dots are associated with colored capital letters, and indicate the selected sampling subenvironment/location. Samples were obtained from orthoquartzite walls (**b**, green dots - Q), quartz sand lying on the cave floor (**c**, red dots - S), opaline silica growing on cave walls (**d**, blue dots - WL), opaline speleothem on the floor (**e**, yellow dots - F) and opaline silica precipitates and slime floating on cave ponds (**f**, white dots - WB). Panel (**g**) shows the degree of silica amorphization, pH and the amount of major and minor elements among the different samples and in cave waters (STR refers to running stream waters; WB refers to standing water ponds). All photos are provided by La Venta Archive (**a**, V. Crobu; **b**–**e**, L. Piccini; **f**, R. De Luca).

**Figure 3:**
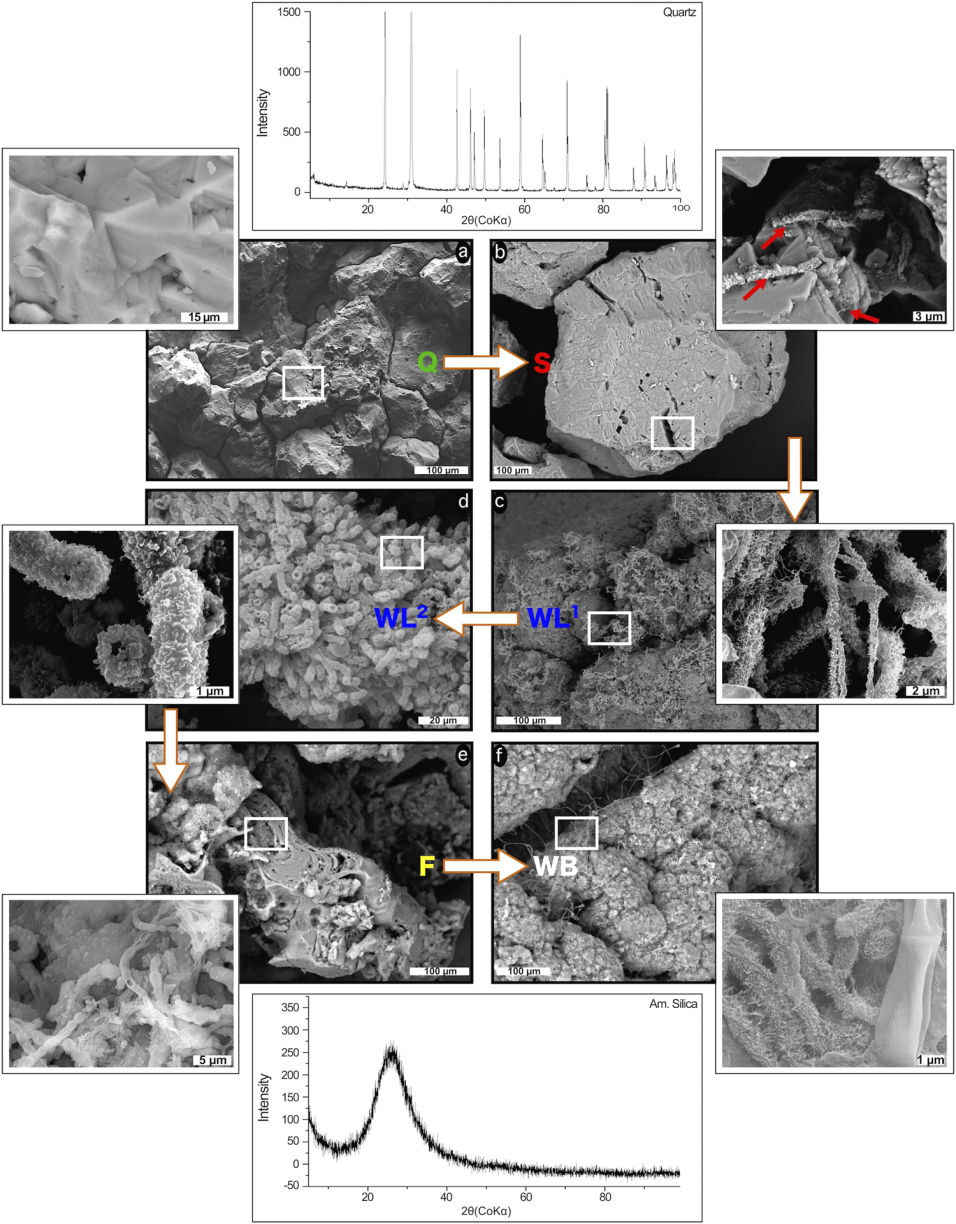
SEM/FESEM images (**a**–**f**) of the samples under analysis, i.e. Q, S, WL (represented by WL1 and WL2), F and WB. White arrows on the images display the proposed progression of the microbial colonization and silica mobilization through the different cave subenvironments represented by each sample. Beside each image, magnifications are shown which enlarge representative areas (within white rectangles).

To describe the silica mobilization processes, different environments within the cave (Supplementary Fig. S1) were analyzed representing subsequent stages of silica demineralization from quartz and precipitation as amorphous silica. Five samples were collected from cave subenvironments in Imawarì Yeuta (Fig. 2a) representing different biogeochemical niches from the unweathered orthoquartzite to the amorphous silica deposits and silica-saturated waters on the orthoquartzite bedrock. Sample Q corresponds to a recently eroded orthoquartzite wall (Fig. 2b), in which degradation produces loose quartz sand that is accumulated on the cave floor (sample S, Fig. 2c). Quartz amorphization is absent in Q and minimal in S with XRD spectra showing a composition of exclusively α-quartz (Fig. 3). Sample WL is a white soft paste of amorphous silica showing a transition from the orthoquartzite wall surface to thick but soft laminated deposits (Fig. 2d). The XRD spectra (Fig. 3) of WL confirmed a pervasive silica amorphization to gel-like opal-AG^19^. Sample F corresponds to a well-consolidated laminated amorphous silica speleothem on the cave floor (Fig. 2e) also composed of opal-AG. WB is from a standing water pool saturated with respect to silica, with evident iridescent violet patinas (Fig. 2f) floating on the surface and amorphous silica and sulfate deposits around the pool edges. SiO_2_ dominates all subenvironments (Fig. 1g), but minor elements, such as iron and aluminium, slightly increase from Q to S and speleothems WL and F (Table S1). pH of moisture wetting the different environments also increases from 4 in Q and S to 5 in the amorphous silica samples (Table S1). A similar trend is shown by cave water chemistry: in active stream waters (STR) silica content is low (0.1–1 mg L^−1^) and pH is acidic (3.5 to 4.5), while standing pool waters (WB) are saturated with respect to silica (>8 mg L^−1^), pH reaches 6 and minor components like sulfates, chlorine and barium are much higher than the stream waters (STR)^12^ (Fig. 1g).

### Microscopy evidences of biologically mediated silica mobilization

Quartz toward Opal-AG transition, occurring through the selected subenvironments, is accompanied by a gradual increase and complexity of ultrastructures related to microbial activity (Fig. 3). Sample Q is built of interlocked quartz grains and quartz overgrowths showing signs of dissolution (V-pit features; Fig. 3a)^4^. Biofilms and amorphous silica precipitation have not been detected, suggesting that silica mobilization is mainly controlled by extremely slow undersaturated water surface-controlled advection and chemical diffusion in the rock porosities, as reported by previous studies on orthoquartzite chemical weathering^10^. V-pits are much more developed in S, and in some places evolve into deep hollows covered by microbe-related short filaments (Fig. 3b, Supplementary Fig. S2). The presence of such biosignatures is accompanied by an increase of dissolution pits, but also by the precipitation of amorphous silica coating tubular-shaped structures around the pits (arrows in Fig. 3b). Bacterial colonization abruptly increases in WL, with areas extremely rich in biological structures, composed of networks of very thin interwoven filaments and spore-like features with appendages (WL1, Fig. 3c, Supplementary Fig. S2). In other areas, filaments are the locus of amorphous silica precipitation, forming botryoidal masses and tubular casts (WL2, Fig. 3d, Supplementary Fig. S2), both completely enveloping the original quartz grains. Finally the quartz grains are completely covered by amorphous silica permineralized microfossils. Similar tubular structures have been observed in other *tepui* caves^6,7^, and volcanic caves^20^, forming highly-porous amorphous silica material having a high capillary water-retaining capacity^14^. The fabric of sample F is much more complex: the amorphous silica is layered and biological structures such as tubular sheets and spore-like chains are completely encrusted by amorphous silica, constituting a compact and dense aggregate (Fig. 3e). Patinas floating on the water body (WB) show similar structures as the interwoven filaments detected in WL1, with local encrustation of amorphous silica most probably representing aggregates of microbial biofilm developed on the water surface (Fig. 3f).

**Figure 4:**
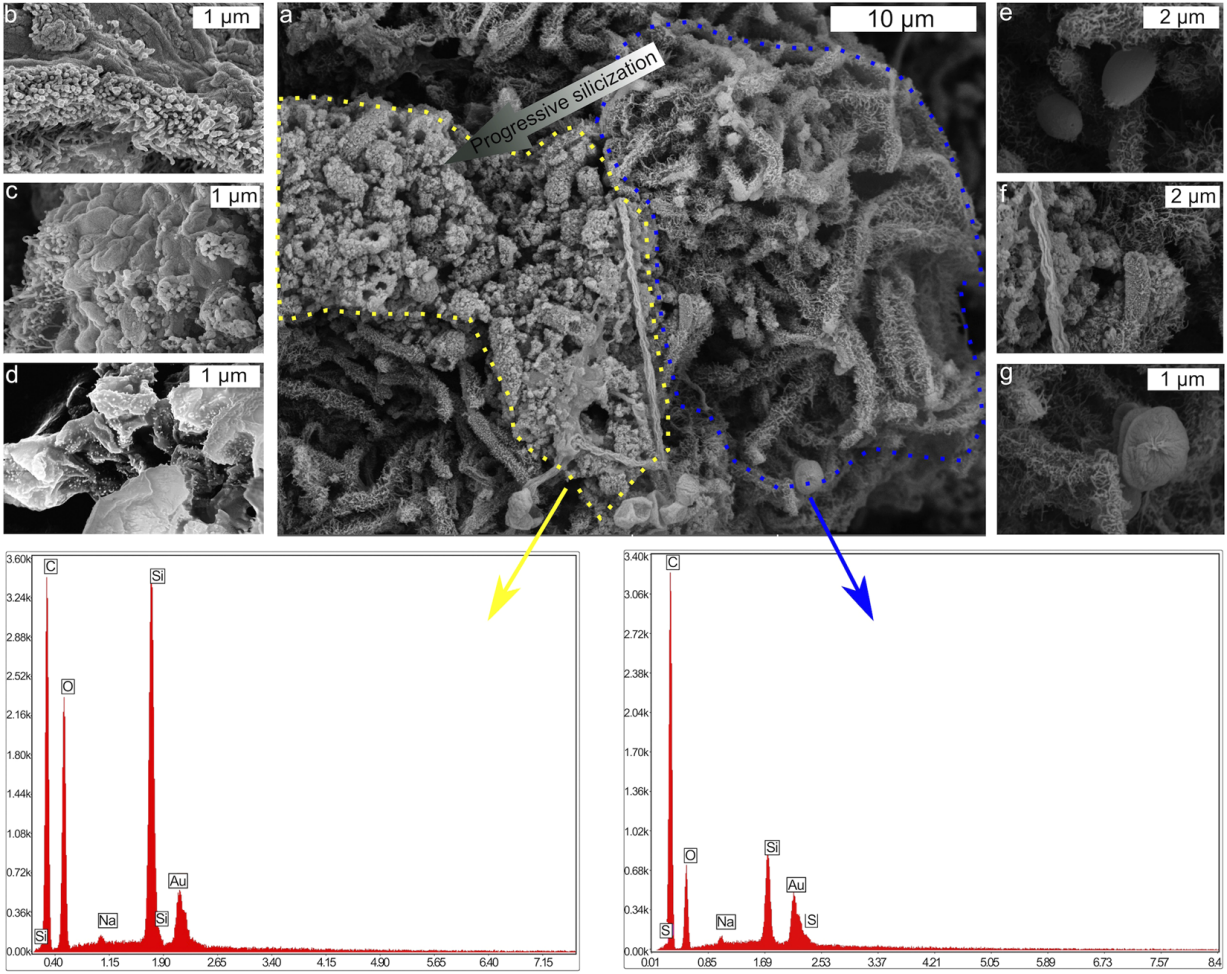
Encrustation of microbial communities by amorphous silica in WL sample. Poorly (blue dotted-line area) and intensely (yellow dotted-line area) encrusted microbial filaments (**a**); besides the morphology, evidences of progressive amorphous silica coating are provided by the EDS patterns shown below. Amorphous silica precipitation mostly occurs on filaments (**a**,**b**), produced by bacillary bacterial cells (**e**–**g**). Deposition of amorphous silica also occurs on biofilms (**c**) and spore-like features (**d**). See also Supplementary Fig. S2.

Sample WL (Fig. 4, Supplementary Fig. S2) shows that the amorphous silica coating is enhanced on bacterial filaments, probably produced by hairy bacillary cells embedded in the filamentous mat (Fig. 4e–g). SiO_2_ precipitation mainly occurs on the exterior part of the interwoven filaments (Fig. 4b), building the wall of the tubular casts. However, amorphous silica also covers biofilm- and spore-like structures (Fig. 4c,d). Different stages of amorphous silica coating, which correspond to specific EDS spectra, can be distinguished (Fig. 4a): where filaments are poorly encrusted, Si is low and C prevails. In the case of highly encrusted filaments, Si and O rise to the level of C evidencing a higher degree of colloidal silica precipitation.

### Microbial community diversity

The Illumina MiSeq sequencing of the five samples collected from Imawarì Yeuta cave generated a total of quality filtered 60,491 sequence reads (301 bp average length) that clustered into a total number of 36,915 OTUs, at a 97% cut-off for sequence identity (Table S2). Around 50–70% of the reads clustered into very low abundant OTUs (with a frequency <0.01% of the total population) (Supplementary Fig. S3).

Hierarchical UPGMA trees, based on Pairwise Bray-Curtis distance, clustered the samples Q and S together, both considering the OTU level and the RDP classification (Supplementary Fig. S4). The relatedness of this cluster with the sample WL and the separation of WB and F indicated more similar bacterial composition in samples collected from the cave wall compared to those collected from the cave floor and water body. Samples Q, S, and WL also showed higher bacterial diversity compared to the samples collected from the cave floor and water body, on the basis of Shannon and Simpson indexes (Table S2). Despite the higher similarity among the wall-related samples, a low percentage of OTUs is still shared among them (<2%), while, in general, less than 1% of the total number of OTUs recovered was found in any of the sampled communities (Supplementary Fig. S5).

### Microbial community taxonomic composition

**Figure 5:**
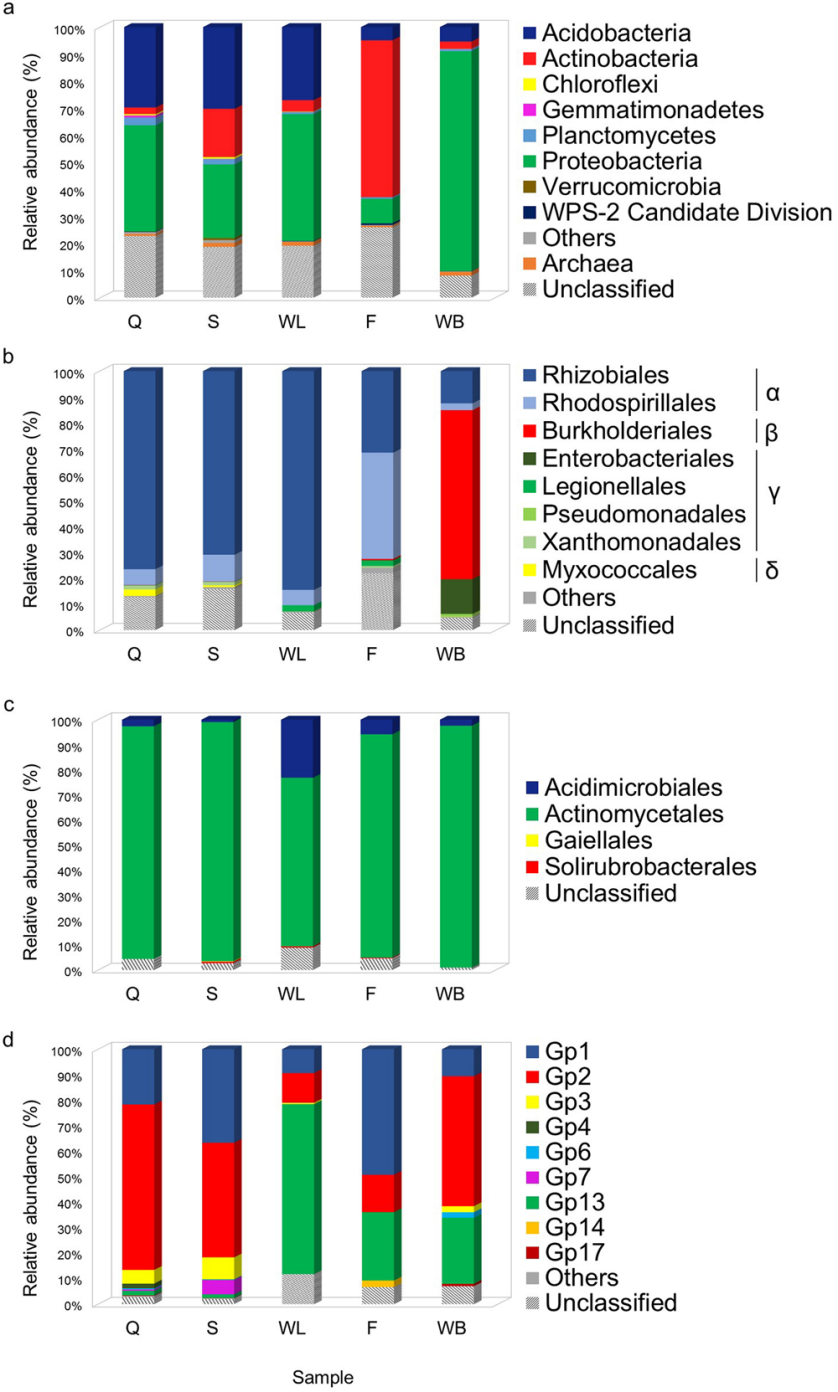
Microbial community composition for the Imawarì Yeuta cave samples representing progressive stages of silica precipitation. Classification was performed using the RDP Classifier provided with the function of 16S rRNA gene copy number adjustment (data from Tables S3–S7). Sequences that could not be classified by RDP with more than 80% of similarity to reference sequences were determined as unclassified. (**a**) Distribution of bacterial phyla and Archaea in cave samples. The category “Others” represents bacterial phyla that constitute less than 0.5% in all samples and includes the phyla *Armatimonadetes*, *Bacteroidetes, Chlamidiae, Cyanobacteria/Chloroplasts, Firmicutes, Fusobacteria, Nitrospirae, Thermotogae, WPS-1 candidate division*. (**b**) Distribution of *Proteobacteria* (order). Proteobacterial classes α (alpha-), β (beta-), γ (gamma-) and δ (delta-) *proteobacteria* are indicated on the side of the corresponding orders. The category “Others” represents *Proteobacteria* orders that are <0.5% in all samples and includes the orders *Alteromonadales, Bdellovibrionales, Caulobacterales, Desulphovibrionales, Ferrovales, Hydrogenophilales, Nitrosomonadales, Oceanospirillales, Rhodobacterales, Rhodocyclales*. (**c**) Distribution of *Actinobacteria* (order). (**d**) Distribution of *Acidobacteria* groups. The category “Others” represents *Acidobacteria* groups that are <0.5% in all samples and includes the groups Gp5, Gp10, Gp11, Gp12.

A high portion (>70%) of the sequences obtained from the five Imawarì Yeuta samples remained unclassified at family and genus level, whereas <25% of the microbial communities resulted unclassified at phylum level (Fig. 5, Tables S3–S7). A total number of 17 eubacterial phyla were identified, with 16 phyla present in Q and S, 11 phyla in WL, and 8 phyla in F and WB. The three phyla *Proteobacteria*, *Actinobacteria*, and *Acidobacteria* represented 75–80% of each microbial population with relative abundances varying among the samples (Fig. 5a). Additional phyla present in all samples were *Planctomycetes* and *Chloroflexi*, which were ≥2% and >0.5% only in Q and S, respectively, and the Candidate Division WPS-2, with a maximum abundance of 0.6% in F. Other low abundant phyla (>0.5–1%) present in selected samples were *Gemmatimonadetes*, which was detected in Q (0.7%), S and WB (0.1% each) and *Verrucomicrobia*, which was found in S (0.8%), Q and WL (0.1% each). Sequences belonging to *Archaea*, despite being identified in all the samples, were detected with low abundance (0.8–1.5%) (Fig. 5a, Tables S3–S7).

Within Q, S, and WL, *Proteobacteria* were dominated by the *Alphaproteobacteria* class mainly composed of *Rhizobiales* (70–80%) and, at a lower percentage, of *Rhodospirillales* (6–10%) (Fig. 5b). Despite their relationship at phylum level, some peculiar differences were found among the wall-related samples including: (i) a variety in *Acidobacteria* groups featuring each sample (Gp2 in Q, both Gp1 and Gp2 in S, and Gp13 in WL); (ii) a higher abundance of *Actinobacteria* in S representing around 20% of the total microbial community, while representing <5% in Q and WL; (iii) a decrease in *Deltaproteobacteria* (mainly constituted of *Myxococcales*) and *Planctomycetes* going from Q to S and to WL; iv) a reduction of the amount of identified bacterial phyla (Fig. 5, Tables S3–S7).

In sample F *Actinobacteria* represented almost 60% of the microbial population constituted of *Actinomycetales* (89%) and *Acidimicrobiales* (6%) (Fig. 5c). The *Proteobacteria* were <10% in F and mainly constituted by *Alphaproteobacteria Rhodospirillales* followed by *Rhizobiales* (Fig. 5b). *Acidobacteria* constituted <5% of the microbial community in F with Gp1 being the dominant *Acidobacteria* group followed by Gp13, Gp2 and the peculiar presence of Gp14 group (Fig. 5d).

More than 80% of the microbial community of sample WB was represented by *Proteobacteria* mainly composed of *Betaproteobacteria* (65%) classified as members of *Burkholderiales* order and *Janthinobacterium* genus (Fig. 5b, Table S7). Further, *Proteobacteria* phylum in sample WB showed a higher presence of *Gammaproteobacteria* (15% of the *Proteobacteria*-related reads) compared to samples Q, S and WL (Fig. 5b). *Actinobacteria* and *Acidobacteria* in WB were as low as 2.6% and 5.3%, respectively, with *Acidobacteria*-related sequences dominated by Gp2 followed by Gp13 and Gp1 (Fig. 5d).

**Figure 6:**
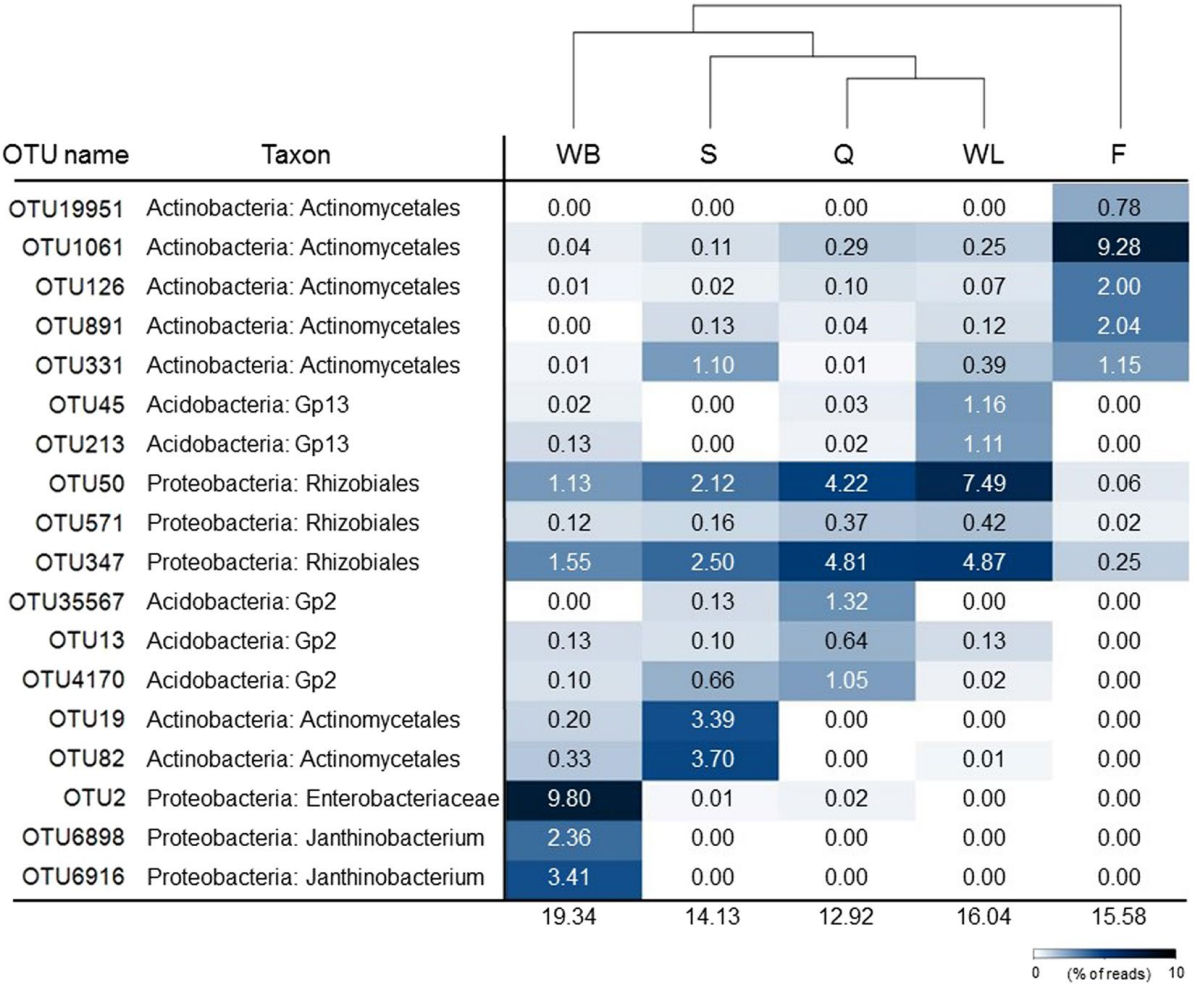
Heat map showing the relative abundance of the 5 dominant operational taxonomic units (OTUs) in each sample. Taxa were defined by using RDP classifier with 80% selected as threshold. Numbers show the relative abundance (% of the reads) of each OTU within the microbial community of each sample. Samples Q and WL are clustered in terms of dominant OTUs and are closely related to sample S. Sample F clusters separately indicating a strong diversity in the dominant OTUs compared to the other samples.

**Figure 7:**
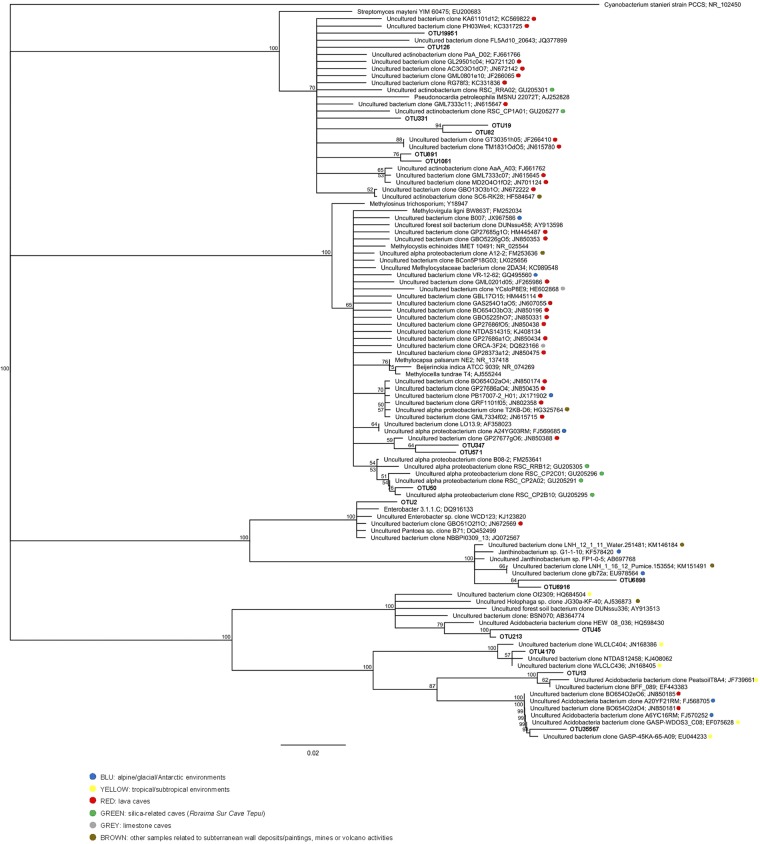
Neighbor-joining tree of the bacterial lineages dominant in the Imawarì Yeuta cave samples under analysis. Sequences retrieved from the present study are shown in bold. The topology of the phylogenetic tree was evaluated by bootstrap re-sampling method with 1,000 replicates, and bootstrap values are shown. The *Rhizobiales*- and *Actinobacteria*-related OTUs matched with reference sequences collected from geographically distinct caves including different lava tubes and the only other silica-based cave previously studied in the *tepui* area (Roraima Sur Cave). Further Imawarì Yeuta cave dominant OTUs clustered with sequences from ancient subterranean Etruscan paintings and extreme environments such as alpine and Antarctic soils. *Janthinobacterium*-related OTUs also clustered with reference sequences detected in a vulcano-generated habitat on a lake surface in Chile.

The dominant OTU-based clustering analysis indicated that the wall-related samples were closely related in terms of dominant lineages, while F clustered separately from the other samples because of the strict dominance of *Actinomycetales*-related sequences and the absence of OTUs that were dominant in the other samples (Fig. 6). In general, high abundant OTUs constituted less than 20% of each microbial community ranging from a minimal value of 13% in Q to a maximum of 19% in WB (Fig. 6). The reference sequences in Genbank that shared high similarity (>96%) with the Imawarì Yeuta dominant OTUs were recovered from i) different cave systems with distinct origin and geographical localization, ii) environments featured by glacier/antartic or tropical/subtropical temperatures, iii) other peculiar ecosystems like a vulcano-generated habitat in Chile and two heavy-metal contaminated sites (Fig. 7). Two OTUs affiliated with the *Rhizobiales* order (OTU50 and OTU347) were predominant in Q and WL and present in samples S. Their representative sequences were phylogenetically related (98% of sequence identity) to reference sequences of members of *Beijerinckiaceae* and *Methylocystaceae* (Figs 6 and 7). Additional abundant OTUs in sample Q were classified as *Acidobacteria* Gp2 and in sample WL as *Acidobacteria* Gp13. In sample S, the predominant OTUs were classified as *Actinomycetales* and were very low abundant or absent in the other samples. Distinct OTUs belonging to *Actinomycetales* were predominant in sample F with OTU1061 representing almost 10% of the library. With the same percentage, *Enterobacteriaceae*-related OTU2 was predominant in sample WB followed by two OTUs belonging to *Janthinobacterium* genus (Figs 6 and 7).

Most of the reference sequences, within the *Actinobacteria* and *Alphaproteobacteria* in Fig. 7, derived from the characterization of microbial mats collected from European and American lava caves^20,21^. *Actinomycetales*-related OTU331 and *Rhizobiales*-related OTU50 shared also high similarity (97% and 99%, respectively) with reference sequences recovered from Roraima Sur cave that is the only other quarzite cave microbiologically described^22^. Among *Enterobacteriaceae* and *Acidobacteria*, OTU2 and OTU35567 also shared high similarity (99%) with sequences collected from tan and white microbial mats, respectively, recovered from a lava cave in Azores^20,23^. The two *Janthinobacerium*-related OTU6898 and OTU6916 shared a similarity of 98% with two sequences recovered from a volcano-generated habitat composed of a silica pumice substrate floating on a lake surface^24^.

## Discussion

Orthoquartzites are among the less soluble rocks in the Earth’s crust. Accordingly, the formation of giant caves, extensive weathering features, and significant amorphous silica deposits in orthoquartzite environments appear as an unresolved paradox because of the extremely slow solution kinetics of quartz in low temperature and neutral-acidic pH conditions^4,13^. In order to find an answer to this puzzle, the direct role of microorganisms in silica mobilization and precipitation processes has been widely debated as one of the most likely and mostly unknown factors involved^9,14,25^. In this work, the Illumina sequencing combined with the geochemistry and microscopy analyses of different samples provided insights into the microbial diversity featuring the consecutive stages of silica amorphization. Although the 16S rRNA gene-based analysis does not describe microbial functional traits, the presence of specific microbial taxonomic groups along with the detection of biosignatures as elemental composition variations and peculiar microscopic structures gave indications on the putative metabolic activities involved in silica mobilization and silica-based speleothem formation in the subsurface^6,26^.

The first evidence of the microbial role in silica amorphization process in Imawarì Yeuta cave was provided by secondary electron microscopy analysis that highlighted the presence of tubular casts and filamentous structures ascribable to the silification of microbial cells and metabolic products (e.g. EPS, biofilm). These observations are in line with previous studies showing that the precipitation of amorphous silica colloids and gels is enhanced on microbial cell surfaces with their ultrastructures and extracellular polymeric substances (EPS) acting as nucleation sites, even when the aqueous solutions are apparently undersaturated with respect to the orthosilicic acid^27,28^. In our samples, the complexity of the structures and the level of silicification increased progressing from Q towards F and WB, which represent the mature stages of silica amorphization process in floor and water body.

Some aggregates of filamentous structures and permineralized tubular casts share strong similarities with silica precipitates and silica-based peloids found in speleothems from other orthoquartzite caves of the *tepuis*^14^. In this previous research, silica precipitation and speleothem formation were ascribed to the activity of heterotrophic or autotrophic filamentous bacteria like cyanobacteria, by analogy with microorganisms associated with modern silica stromatolite communities in hydrothermal sinters^29^. Conversely, our present results attest only traces (<0.05%) of cyanobacteria (found exclusively in Q and S, Tables S3 and S4), discrediting their role in silica mobilization at least in the Imawarì Yeuta cave samples. On the other hand, the silicified tubular structures and interwoven filaments observed in the opaline speleothems under analysis also share strong similarities with silica precipitates found in low-temperature hydrothermal fields^27^. In these latter environments, some phylotypes related to Fe-oxidizing bacteria were detected and ultrastructures related to FeOBs activity, visible by microscope, were proposed to serve as nucleation template and scaffolding for silica accumulation and precipitation^27^. In the case of Imawarì Yeuta cave, possible Fe-oxidizing bacterial groups are included in predominant phyla detected in the cave i.e. *Acidobacteria*, *Proteobacteria*, and *Actinobacteria* phyla. Members of *Alphaproteobacteria* such as *Methylocella* genus were described to have Fe-oxidizing activity as well as *Actinobacteria* of the *Acidimicrobiales* groups^30,31^. A few OTUs related to specific Fe-oxidizing betaproteobacterial genera (*Thiobacillus* of *Hydrogenophilales* order, *Ferrovum* of *Ferrovales* order, and *Cupriavidus* of *Burkholderiales* order) were detected (Tables S3–S7). The occurrence of Fe-related microbial activities is also supported by the increase, throughout the amorphization process, of iron concentration from the quartz samples (Q and S) to the amorphous silica speleothems (WL and F) (Fig. 1g; Table S1). The presence in all samples of bacterial groups with possible Fe-oxidizing activities makes Fe-oxidizers potential candidates involved in the formation of amorphous silica speleothem.

The geochemical analysis also indicated the peculiar increase of metals, other than Fe, along with minor elements during the amorphous silica deposition. This suggests that additional biomineralization processes could be involved in silica mobilization. Indeed, in such an oligotrophic environment, bacterial communities are expected to take advantage of element diffusion from the orthoquartzite, which concurrently involves not only silica but also other minor rock components^13^, such as iron, zinc, barium and calcium which are necessary for the microbial metabolism/growth. Similar processes of metal mobilization from the host rock have been described also in hypogenic caves in limestone^32,33^ and in peculiar iron-silica caves in Brazil^34^. In these cases the microbial community is proposed to bio-weather the rock substrate for accessing reduced metals (mainly manganese and iron) that are oxidized by microbial activities and deposited at the rock-air interface (i.e. walls and floors of the cave).

The increase of Ba^2+^ detected in WL and F, and in the standing water pool WB represents an interesting feature of Imawarì Yeuta cave^12^. At near neutral pH conditions, even a limited concentration of Ba^2+^ in solution enhances the dissolution rate and solubility of quartz as much as forty times as compared to deionized water, having a strong influence on the overall silica mobilization potential^35^. Bacterial ability to mobilize, concentrate and precipitate barium compounds was demonstrated using bacterial isolates^36^, while microbial biofilms were shown to play a role in the formation of barium-containing deposits found on volcanic rocks in catacombs^37^. Recently, biomineralization of barium was also found to occur intracellularly in filamentous bacteria symbiotic of marine silica sponges^38^, suggesting a potential direct role of barium in the biotic control of silica precipitation. In consideration of the environmental conditions within Imawarì Yeuta cave, microbes are likely to have a role in barium precipitation through biomineralization processes^37^, or through bioaccumulating Ba in extracellular polymeric substances (EPS) and/or in cell walls functioning as nucleation sites^36,39^. Possible metal-oxidizing microbial activities are related to *Janthinobacterium* spp. present in WB, to members of *Actinomycetales* in F and of *Rhizobiales* in wall-related samples. Previous studies have also indicated members of *Rhizobiales* and *Actinobacteria* to be involved in biomineralization processes and rock weathering in cave environments^20^, while a *Janthinobacterium* strain was described to perform Mn oxidation after being isolated from cave ferromanganese deposits^40^. Taken together, these considerations support the conclusion that the tubular and filamentous structures observed in speleothems of Imawarì Yeuta and related to uneven amorphous silica precipitation are likely due to biologically-driven processing of various elements.

The taxonomy analysis of the Illumina sequencing data revealed that the wall-related samples Q, S, and WL had a higher bacterial diversity and a more similar microbial community composition as compared to F and WB (Tables S2–S7, Supplementary Fig. S4). In particular, the wall-related microbial communities were dominated by *Alphaproteobacteria* (mainly *Rhizobiales*) and *Acidobacteria* while the samples collected from the cave floor and water body (F and WB) were characterized by *Actinobacteria* (mainly *Actinomycetales*) and *Betaproteobacteria* such as *Janthinobacterium*, respectively. Within *Alphaproteobacteria*, members of the *Beijerinckiaceae* and *Methylocystaceae* families of *Rhizobiales* order were highly abundant in Q and WL which include the genera *Methylocella* and *Methylocystis* able to fix nitrogen and metabolize C_1_-compounds^41^. In the same way, although the knowledge on their metabolic function in caves is still limited and a high variation was described among members of this phylum, some *Acidobacteria* presented genomic traits correlated with oligotrophy supporting an ecological advantage when low inputs of organic matter are available^42^. Possible metabolic interpretations deriving from the microbial diversity described in the samples collected from the wall (Q and WL) suggest the presence of chemolithotrophic bacteria able to generate the primary production, which supports the sustenance of complex microbial communities under the oligotrophic conditions featuring the orthoquarzitic cave wall-samples. The silica speleothem evolution on the wall was parallel to bacterial groups diversification moving from Q to WL, including a variation in *Acidobacteria* groups and a decrease in *Deltaproteobacteria* and *Planctomycetes* (Fig. 5, Tables S3–S7). These microbial composition changes were more dramatic moving from the wall samples to F and WB, where the increase of the amorphization of the silica is also parallel to a possible increase of organic matter input associated to external sources (water flowing from outside, air flows, and cave fauna). In these samples, the high abundance of *Actinomycetales* in F and *Bukholderiaceae* and *Gammaproteobacteria* in WB could be associated to biomineralization processes possibly associated to the variation in elemental composition detected through geochemical analyses and/or to the filamentous structures visible through microscopic analyses^20,23^. On the other hand, the sample S that derives from the erosion of Q presents bacterial profile dominance similar to the wall samples and a silica amorphization stage that seems to be in between Q and WL, although the influence of the floor location on S microbial community is highlighted by the increase of *Actinobacteria* and the presence of *Actinomycetales* among the most abundant phylotypes. We therefore propose that there is a mutual influence between the silica amorphization progress and the microbial population composition, which is driven by both the nature of the nutrient inputs and the geochemistry of the microenvironments, the nature of these aspects in turn being related to the sampling site and the silica solubilisation process. In this regard, a consecutive increase of the metal ions concentration and inorganic cations as well as pH alkalinisation were parallel to silica amorphization in Imawarì Yeuta speleothems. Local changes in pH and the production of metabolites (e.g. EPS and amino acids) that influence silica solubility can result from bacterial metabolic processes related to chemolitotrophic activities, e.g. CO_2_ fixation and inorganic nitrogen transformation^43,44^. In this respect, the wall-related samples showed a high abundance of microorganisms able to perform N_2_ fixation and C_1_ compound metabolism such as *Beijerinckiaceae* and *Methylocystaceae* members of *Rhizobiales*. Low abundance of microorganisms like the ammonia oxidizer *Nitrosomonas* and the nitrite-oxidizing *Nitrospirae* and *Nitrobacter* were detected in all the Imawari Yeuta samples (Tables S3–S7). These bacterial groups also include members able to degrade urea into ammonia and CO_2_ and their presence might be correlated to CO_2_-fixation-coupled ammonia oxidation processes^45^. Further, in relation with the pH shift observed during silica speleothem formation, diverse *Acidobacteria* groups characterized each silica mobilization stage on the wall, suggesting a specific contribution to the diverse microscopic morphologies and/or a different response to the pH change and geochemical composition. On the other hand, members of the *Actinobacteria* phylum dominated samples localized on the cave floor, i.e. S and F. Most of them are heterotrophic, feeding on organic carbon, but some are also known to fix nitrogen and to have chemolithoautotrophic activities exhibiting nitrate-dependent iron oxidation^20^.

**Figure 8:**
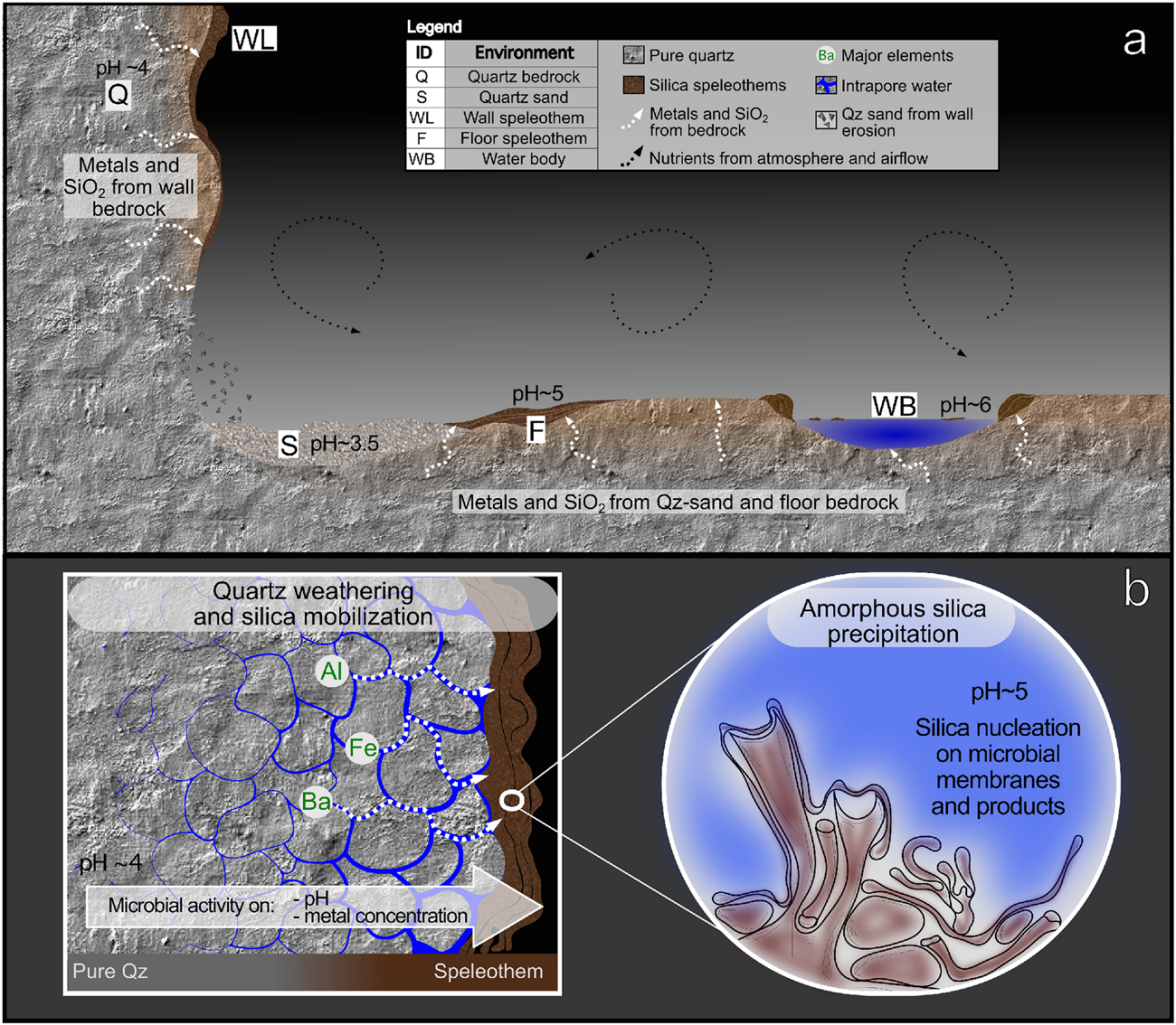
Schematic representation of the Imawarì Yeuta cave (**a**) and the processes of silica mobilization and precipitation (**b**) leading to the formation of biogenic silica deposits in *tepui* caves.

Taken together, our results indicate that complex chemotrophic microbial communities colonize different niches in the cave and create the chemical conditions driving quartz dissolution through i) the increase of the amount of inorganic cations and metal ions in solution as a result of biomineralization processes; ii) the raise of pH mediated by microbial metabolisms (e.g. nitrogen fixation, decomposition of proteins or amino acids, urea degradation, CO_2_ consumption). Silica solubilized from the rock can reprecipitate as amorphous species on microbial cell surfaces with their ultrastructures and extracellular polymeric substances (EPS) acting as nucleation sites as observed in Fig. 4. Biologically-mediated silica dissolution and reprecipitation in turn can lead to new silica mobilization from the rock by boosting further chemical diffusion. Figure 8 shows a working model of the mechanisms we propose are involved in the microbial-mediated silica solubilisation and precipitation in Imawarì Yeuta cave.

This research shows that the mobilization of important amounts of silica can occur not only in hydrothermal conditions but also in non-thermal subsurface niches such as orthoquartzitic caves. In particular, the analyses of Imawarì Yeuta cave samples revealed the presence of specific microbial groups, microbial-like microscopic structures and peculiar variation of elemental composition (e.g. Ba^2+^), which support the role of complex chemotrophic bacterial communities in silica mobilization and silica speleothem formation. Our finding not only opens new perspectives on the study of silica mobility in natural environments, but also raises questions on the possibility that some siliceous Precambrian microfossils and stromatolites might have formed through similar biologically-mediated mechanisms, different from those occurring under hydrothermal conditions and different from those mediated by photosynthetic organisms^8^. This discovery provides new insights into the relationship between silica and biologically-mediated precipitation processes and into the definition of novel biosignatures in silica-rich deposits.

## Material and Methods

### Sampling sites and collection methods

Five different rocky surfaces/deposits were sampled with the aim to represent the majority of cave hydrological environments and biological niches related to silica mobilization processes (Fig. 2).

After scraping/collection with sterilized tools, all samples were stored in Eppendorf tubes filled with a solution of LifeGuard RNA. The transport from the site to the lab was carried out in a portable fridge, then samples were stored at −80 °C until analysis.

### X-Ray fluorescence spectrometry

Bulk chemical analyses were conducted by a wave dispersive X-ray fluorescence spectrometer (WD-XRF) operating at BIGEA department, University of Bologna (Italy). Ultra-fine powdered samples were mounted on rounded boric acid casts (~5 cm diameter, ~0.5 cm height), which were prepared according with the matrix correction method^46–48^. Thirty-five international reference materials were used for calibrating the raw results, allowing an accuracy better than 5% for elements >10 ppm, and between 10% and 15% for elements <10 ppm. Thermogravimetric TG–DTG–DTA measurements were performed by using a Setaram Labsys double-furnace apparatus and calcined Al_2_O_3_ as reference substance, in order to calculate the volatile content. Powdered 0.5 g samples were placed in platinum crucibles and introduced into the furnace at 800 ± 1 °C for ~24 hours drying before the final weighing.

### Water chemical analyses

Water temperature (T) and acidity (pH) were measured by handheld field instruments (Hanna Instruments) after calibration on site. Accuracy was 0.1 °C and 0.01. pH on water films on orthorquartzite surfaces (Q), quartz sand (S) and amorphous silica speleothems surfaces (WL and F) was measured with pH stripes with range 2 to 9 and 0.5 pH unit increments (Macherey Nagel 92118). Dissolved silica concentration (DSi) was measured by using a field colorimetric test kit (Aquaquant 14410 Silicon - Merck), that allows the determination of silica in the concentration range 0.01–0.25 mg L^−1^ with an error less than 20%. Samples with concentration higher than 0.25 mg L^−1^ were diluted with distilled water and then analyzed. Results were expressed following the convention of representing dissolved silica as the oxide SiO_2_. In order to determine dissolved elements through ICP-MS analyses in the laboratory, double water samples were collected in streams and ponds at Imawarì Yeuta in March 2013: a 250 mL bottle of untreated and unfiltered water, and a 100 mL bottle of 0.45 micron-filtered and 1 mL 65% HNO_3_ acid-preserved water.

Inductively coupled plasma-mass spectrometry (ICP-MS) (method EPA 6020 A) was applied for determination of multi-elemental sub μg L^−1^ concentrations (Al, Sb, As, Ba, Cd, Ca, Fe, Mg, Pb, K, Na, Zn) where the recovery of the Laboratory Control Sample (LCS) resulted between 85 and 115%, as expected by the method lines. Anion Chromatography (method EPA 9056 A) was used to determine chloride, fluoride, nitrate, and sulfate in the solution. NH_4_ concentration was measured on the untreated sample with the method APAT CNR IRSA 4030 A2 MAN 29 2003. Analyses were carried out as in^12^.

### X-Ray diffraction

Mineral phases were investigated by a Philips PW3710 X-Ray diffractometer (current: 20 mA, voltage: 40 kV, range 2θ: 5–80°, step size: 0.02° 2θ, time per step: 2 sec) at the University of Genova (Italy), which mounted a Co-anode, as in^49^. Acquisition and processing of data was carried out using the Philips High Score software package.

### Scanning electron microscope (SEM)

For scanning microscope analyses, subsamples were first covered with a thin evaporated gold layer by sputtering, then introduced into a Vega3 Tescan scanning electron microscope (SEM) and a Zeiss Supra 40 VP field emission scanning electron microscopy (FESEM), operating at the DISTAV department, University of Genova^49^. The first operated at 20 kV and was equipped with an EDAX-Apollo-X DPP3 energy-dispersive (EDS) X-Ray spectrometer, which was applied for major elements spectrometric measurements. Manganese Resolution of Kα = 126 eV allowed the detection of chemical elements heavier than Boron (atomic number greater than 5). Acquisition and elaboration of data was performed by the TEAM Enhanced Version V4.2.2 EDS software. For FESEM images, we used accelerating voltages from 10 Å to 20 kV.

### Total DNA extraction and Illumina sequencing

The samples were extracted for their total DNA using the UltraCleanH Soil DNA Isolation Kit (MoBio, Carlsbad, USA) with slight modifications as previously described^50^. To provide amplicon for Illumina MiSeq analysis, the total DNA was amplified for the V4-V5 hypervariable region of 16S rRNA gene with universal forward 515 F (5′-Illumina overhang-GTGYCAGCMGCCGCGGTA-3′) and reverse 907 R (5′-Illumina overhang-CCGTCAATTCMTTTRAGTTT-3′) primers (IDT DNA Technologies). One µL of total DNA was added to a 50 µL (final volume) PCR reaction mixture containing 25 µL of Premix F (Epicentre Biotechnologies, WI, USA), 200 mM (each) forward and reverse primers, and 0.5 U of Ex *Taq* DNA polymerase (Takara Bio, Japan)^50^. Amplification reactions were carried out under the following thermocycling conditions: 95 °C for 3 min, 30 cycles of 95 °C for 30 s, 55 °C for 30 s, 72 °C for 30 s, with a final extension at 72 °C for 5 min^50^.

PCR amplicons were confirmed by electrophoresis with a 1% (w/v) agarose gel and then purified by AMPure XP beads (Beckman Coulter) prior to the index PCR. Nextera XT Index was incorporated into each of the individual samples during PCR. The thermal cycling program included a first denaturation step at 95 °C for 3 min, followed by 8 cycles of denaturation at 95 °C for 30 s, annealing at 55 °C for 30 s, elongation at 72 °C for 30 s, with a final extension at 72 °C for 5 min. Purified amplicons were submitted to KAUST Genomic Core Lab (https://corelabs.kaust.edu.sa/) for unidirectional sequencing reads on an Illumina MiSeq platform. Sample information and sequences were deposited in the Sequence Read Archive of NCBI under accession number PRJEB22946.

### Sequence analysis and microbial community comparison

Raw sequence reads were first trimmed for the indexes and primer sequences. Trimmed sequences were then checked for their quality by removing reads that are <250 nt in length and with Phred score <20. Chimeras were identified and deleted as previously described^50^. RDP Classifier was used for taxonomical assignments of the 16S rRNA gene sequences at 80% confidence level provided with the function of 16S rRNA gene copy number adjustment. To further perform an OTU-based analysis, all chimera-removed fasta files were combined together with an in-house written Pearl Script. The combined sequence file was then identified for the unique OTUs at 97% 16S rRNA gene similarity using CD-Hit as reported by^51^. The output file denotes the abundance of all unique OTUs in each sample, and the nucleotide sequence of each unique OTU. Taxonomic placement of each OTU was carried out with RDP Classifier^52^. The relative abundance of each OTU was calculated, collated and the normalized data were square-root transformed^50^. The transformed dataset was then computed for their Bray-Curtis similarities and differences in the bacterial communities among the 5 samples were performed by a hierarchical cluster tree created using the unweighted pair-group method with arithmetic mean (UPGMA) with Primer E version 7.

The phylogenetic tree of the most abundant OTUs was performed using the GenBank best hits for each OTU and using Geneious Tree Builder with the Juke-Cantor genetic distance model and the neighbor- joining method. Bootstrap support was calculated (1000 replications).

## Electronic supplementary material

Supplementary Information
