# Supplementary material for: Microbial diversity and biosignatures of amorphous silica deposits in orthoquartzite caves
Source: Sci Rep. 2018 Dec 4;8:17569. doi: 10.1038/s41598-018-35532-y (PMC6279750; doi:10.1038/s41598-018-35532-y)
Supplement: Supplementary file 1 — Supplementary Information [file 41598_2018_35532_MOESM1_ESM.docx]

Supplementary Information of

**Microbial diversity and biosignatures of amorphous silica deposits in orthoquartzite caves**

Francesco Sauro. Martina Cappelletti^*^. Daniele Ghezzi. Andrea Columbu. Pei-Ying Hong. Hosam Mamoon Zowawi. Cristina Carbone. Leonardo Piccini. Freddy Vergara. Davide Zannoni. Jo De Waele

*Martina Cappelletti

Email: [martina.cappelletti2@unibo.it](mailto:martina.cappelletti2@unibo.it)


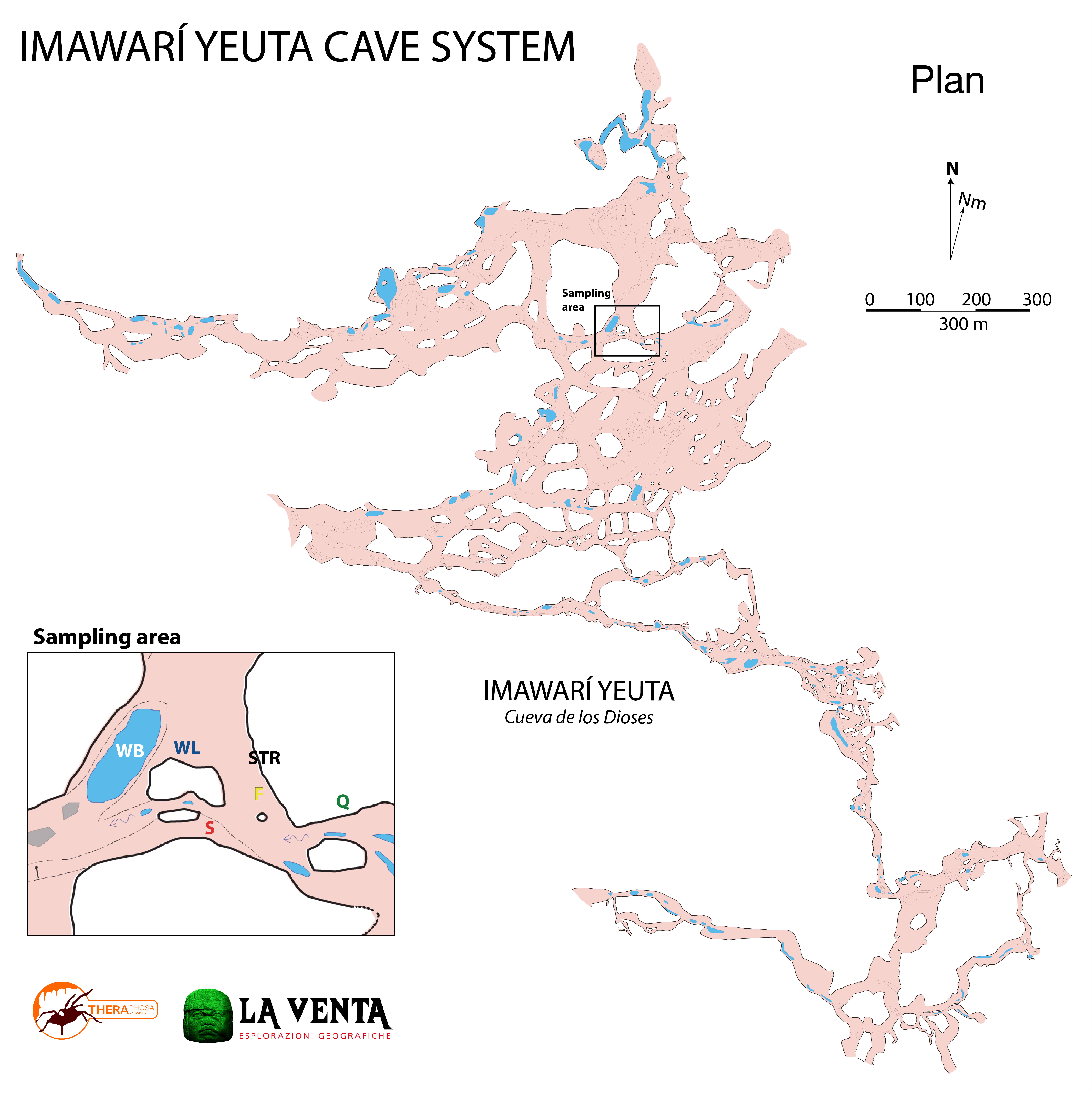


**Supplementary Figure S1**. Plan view of the Imawarì Yeuta Cave System with indicated the sampling area for this study (map credits La Venta-Theraphosa 2016).


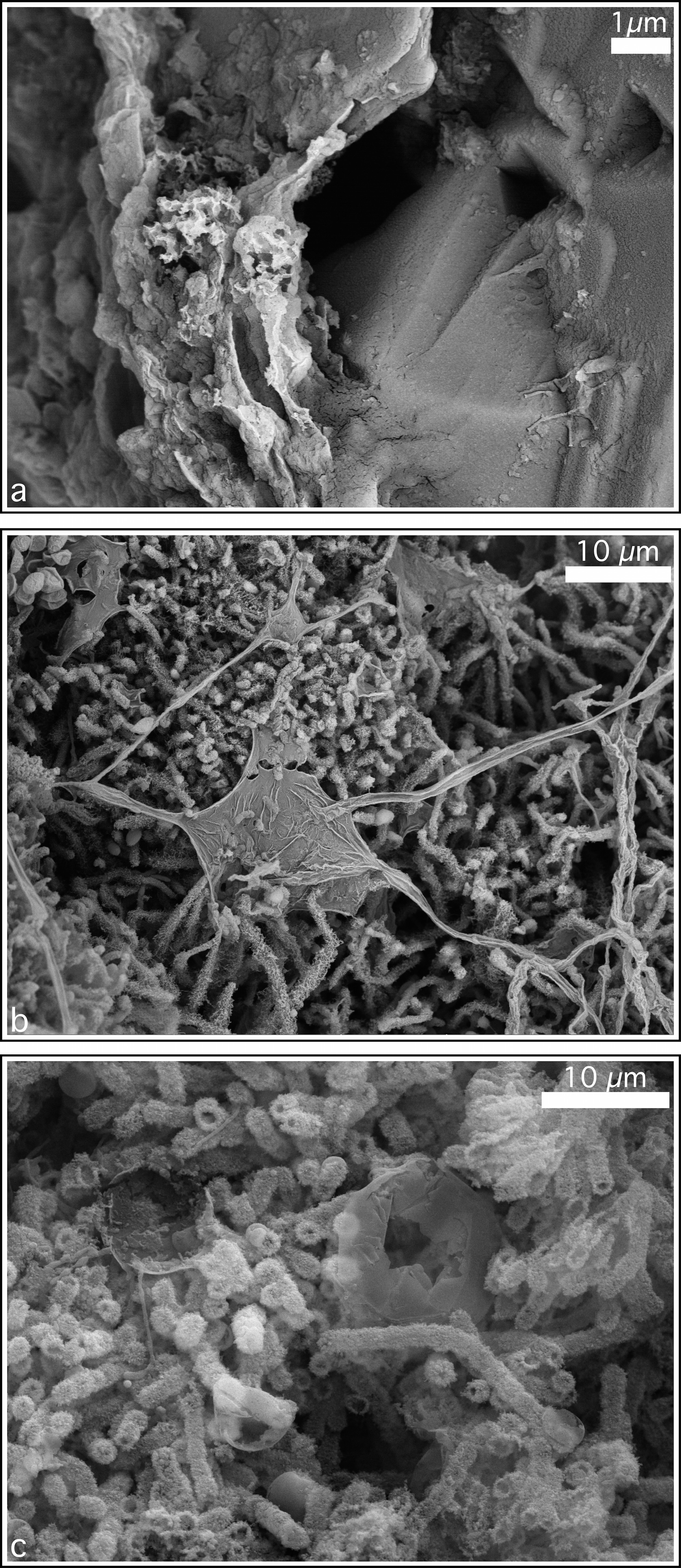


Supplementary Figure S2. SEM images of silicized biofilms and biologic structures in S and WL. a) A quartz grain in sample S characterized by a deep V-pit due to transport controlled dissolution of quartz partially covered by a thick biofilm mat. b) In sample WL a dense network of partially silicified tubular networks of interwoven filaments features are covered by longer flat filaments and patches; c) Silicified tubular casts.

**Supplementary Figure S3.** Analysis of abundant and rare species among the Imawari Yeuta cave samples. A) Percentage of abundant and rare OTUs against the total number of OTUs. B) Percentage of reads included in the rare and abundant OTUs against the total number of reads.

Supplementary Figure S4. Hierarchical cluster tree created using UPGMA with Bray–Curtis distances calculated on the basis of RDP classification (threshold at 80%) (Panel A) and of the OTU abundance (Panel B).

**Supplementary Figure S5.** Venn diagrams showing the specific and shared OTUs among the cave samples under analysis. A) Shared and specific OTUs among the cave samples under analysis. B) Shared and specific OTUs among the cave wall-related samples. i.e. Q. S. WL. C) Shared and specific abundant OTUs among the cave samples. D) Shared and specific rare OTUs among the cave samples

Table S1. Distribution of major elements in waters (blu) and rock/speleothem samples (grey) from Imawarì Yeuta cave.

| **Sample name** | **SiO_2_ mg/L** | **Al μg/L** | **Fe μg/L** | **K mg/L** | **Ba μg/L** | **Cl mg/L** | **SO_4_ mg/L** | **NH4 mg/L** | **Cu μg/L** | **Ca μg/L** | **Mg μg/L** | **pH** | **T**  **°C** |
| --- | --- | --- | --- | --- | --- | --- | --- | --- | --- | --- | --- | --- | --- |
| STR^a^ | 0.65 | 56 | 66 | 0.57 | 1.14 | / | / | 0.69 | / | / | / | 3.9 | 14.5 |
| WB | 8.60 | / | / | / | 2.82 | 1.15 | 0.63 | / | / | / | / | 6.1 | 14.8 |
| **Sample name** | **SiO_2_ wt%** | **Al wt%** | **Fe wt%** | **K wt%** | **Ba ppm** | **Cl ppm** | **SO_4_ ppm** | **Zn ppm** | **Cu ppm** | **Ca wt%** | **Mg wt%** | **pH** | **T**  **°C** |
| Q | 98.0 | 1.6 | 0.1 | 0.0 | 0 | 11 | 0 | 0 | 16.2 | 0.00 | 0.02 | 4 | 14.9 |
| S | 98.48 | 0.93 | 0.15 | 0 | 0 | / | 0 | 0 | 13.5 | 0.00 | 0.02 | 3.5 | 14.9 |
| WL | 93.9 | 1.6 | 0.7 | 0.05 | 37 | 52 | 295 | 75 | 88 | 0.01 | 0.1 | 5 | 14.9 |
| F | 86.9 | 0.8 | 0.4 | 0.01 | 75 | 150 | 170 | 77 | 23 | 0.03 | 0.04 | 5 | 14.9 |

^a^ STR stands for “stream water”. a sample representative of water flowing through the system (undersatured in silica). For all the other samples refer to Figure 1 in the main text.

/ = “not determined”

Table S2. Summary of Illumina sequencing and calculated diversity indices for each sample

| **Sample name** | **# of qualified reads** | **# OTUs** | **Shannon (H’)** | **Simpson (1-’)** |
| --- | --- | --- | --- | --- |
| Q | 19.875 | 11.822 | 8.26 | 0.9951 |
| S | 13.576 | 8.673 | 8.139 | 0.996 |
| WL | 9.908 | 6.642 | 7.854 | 0.9916 |
| F | 8.695 | 6.016 | 7.753 | 0.9901 |
| WB | 8.437 | 4.953 | 7.287 | 0.9877 |

^a^ Operational taxonomic units (OTUs). Shannon and Simpson indices are calculated at 97% sequence identity.

**Table S3 Microbial diversity from domain to genus level of the sample Q^a^**

| **Domain** | **Phylum** | **Class** | **Order** | **Family** | **Genus** | **# reads^b^** |
| --- | --- | --- | --- | --- | --- | --- |
| Archaea | Crenarchaeota | Thermoprotei | Unclassified |  |  | 16 |
| Archaea | Thaumarchaeota | Nitrososphaerales | Nitrososphaerales | Nitrososphaeraceae | Nitrososphaera | 8 |
| Archaea | Thaumarchaeota | Unclassified |  |  |  | 24 |
| Archaea | Unclassified |  |  |  |  | 20.4 |
| Bacteria | Thermotogae | Thermotogae | Kosmotogales | Kosmotogaceae | Mesotoga | 0.5 |
| Bacteria | Acidobacteria | Acidobacteria_Gp1 | Acidobacteria_Gp1 | Acidobacteria_Gp1 | Bryocella | 0.8 |
| Bacteria | Acidobacteria | Acidobacteria_Gp1 | Acidobacteria_Gp1 | Acidobacteria_Gp1 | Candidatus Koribacter | 35 |
| Bacteria | Acidobacteria | Acidobacteria_Gp1 | Acidobacteria_Gp1 | Acidobacteria_Gp1 | Edaphobacter | 1.77 |
| Bacteria | Acidobacteria | Acidobacteria_Gp1 | Acidobacteria_Gp1 | Acidobacteria_Gp1 | Gp1 | 245 |
| Bacteria | Acidobacteria | Acidobacteria_Gp1 | Unclassified |  |  | 324.8 |
| Bacteria | Acidobacteria | Acidobacteria_Gp2 | Acidobacteria_Gp2 | Acidobacteria_Gp2 | Gp2 | 1818.6 |
| Bacteria | Acidobacteria | Acidobacteria_Gp3 | Acidobacteria_Gp3 | Acidobacteria_Gp3 | Candidatus Solibacter | 5.5 |
| Bacteria | Acidobacteria | Acidobacteria_Gp3 | Acidobacteria_Gp3 | Acidobacteria_Gp3 | Gp3 | 65 |
| Bacteria | Acidobacteria | Acidobacteria_Gp3 | Unclassified |  |  | 79 |
| Bacteria | Acidobacteria | Acidobacteria_Gp4 | Acidobacteria_Gp4 | Acidobacteria_Gp4 | Gp4 | 28.8 |
| Bacteria | Acidobacteria | Acidobacteria_Gp4 | Unclassified |  |  | 23.07 |
| Bacteria | Acidobacteria | Acidobacteria_Gp5 | Acidobacteria_Gp5 | Acidobacteria_Gp5 | Gp5 | 3.2 |
| Bacteria | Acidobacteria | Acidobacteria_Gp6 | Acidobacteria_Gp6 | Acidobacteria_Gp6 | Gp6 | 12.2 |
| Bacteria | Acidobacteria | Acidobacteria_Gp7 | Acidobacteria_Gp7 | Acidobacteria_Gp7 | Gp7 | 15.38 |
| Bacteria | Acidobacteria | Acidobacteria_Gp10 | Acidobacteria_Gp10 | Acidobacteria_Gp10 | Gp10 | 3.8 |
| Bacteria | Acidobacteria | Acidobacteria_Gp11 | Acidobacteria_Gp11 | Acidobacteria_Gp11 | Gp11 | 2.6 |
| Bacteria | Acidobacteria | Acidobacteria_Gp12 | Acidobacteria_Gp12 | Acidobacteria_Gp12 | Gp12 | 3.2 |
| Bacteria | Acidobacteria | Acidobacteria_Gp13 | Acidobacteria_Gp13 | Acidobacteria_Gp13 | Gp13 | 50.6 |
| Bacteria | Acidobacteria | Acidobacteria_Gp17 | Acidobacteria_Gp17 | Acidobacteria_Gp17 | Gp17 | 5.1 |
| Bacteria | Acidobacteria | Unclassified |  |  |  | 77.6 |
| Bacteria | Actinobacteria | Actinobacteria | Acidimicrobiales | Unclassified |  | 6 |
| Bacteria | Actinobacteria | Actinobacteria | Actinomycetales | Unclassified |  | 182.8 |
| Bacteria | Actinobacteria | Actinobacteria | Actinomycetales | Thermomonosporaceae | Unclassified | 2 |
| Bacteria | Actinobacteria | Actinobacteria | Actinomycetales | Catenulisporaceae | Catenulispora | 0.3 |
| Bacteria | Actinobacteria | Actinobacteria | Actinomycetales | Pseudonocardiaceae | Unclassified | 1.6 |
| Bacteria | Actinobacteria | Actinobacteria | Actinomycetales | Microbacteriaceae | Unclassified | 1.2 |
| Bacteria | Actinobacteria | Actinobacteria | Actinomycetales | Microbacteriaceae | Pontimonas | 0.6 |
| Bacteria | Actinobacteria | Actinobacteria | Actinomycetales | Sporichthyaceae | Sporichthya | 5.5 |
| Bacteria | Actinobacteria | Actinobacteria | Actinomycetales | Propionibacteriaceae | Propionibacterium | 1 |
| Bacteria | Actinobacteria | Actinobacteria | Actinomycetales | Mycobacteriaceae | Unclassified | 4.8 |
| Bacteria | Actinobacteria | Actinobacteria | Actinomycetales | Mycobacteriaceae | Mycobacterium | 12.7 |
| Bacteria | Actinobacteria | Actinobacteria | Actinomycetales | Streptomycetaceae | Unclassified | 0.3 |
| Bacteria | Actinobacteria | Actinobacteria | Actinomycetales | Streptomycetaceae | Streptacidiphilus | 0.3 |
| Bacteria | Actinobacteria | Actinobacteria | Actinomycetales | Streptomycetaceae | Kitasatospora | 0.1 |
| Bacteria | Actinobacteria | Actinobacteria | Unclassified |  |  | 9.6 |
| Bacteria | Actinobacteria | Unclassified |  |  |  | 0.5 |
| Bacteria | Armatimonadetes | Armatimonadetes_gp4 | Armatimonadetes_gp4 | Armatimonadetes_gp4 | Armatimonadetes_gp4 | 20.8 |
| Bacteria | Bacteroidetes | Flavobacteriia | Flavobacteriales | Flavobacteriaceae | Unclassified | 1.28 |
| Bacteria | Bacteroidetes | Sphingobacteriia | Sphingobacteriales | Chitinophagaceae | Unclassified | 10.7 |
| Bacteria | Bacteroidetes | Unclassified |  |  |  | 0.7 |
| Bacteria | Candidate division WPS-2 | Candidate division WPS-2 | Candidate division WPS-2 | Candidate division WPS-2 | WPS-2_genera_incertae_sedis | 11.11 |
| Bacteria | Chlamydiae | Chlamydiia | Chlamydiales | Unclassified |  | 0.5 |
| Bacteria | Chlamydiae | Chlamydiia | Chlamydiales | Parachlamydiaceae | Unclassified | 0.67 |
| Bacteria | Chlamydiae | Chlamydiia | Chlamydiales | Simkaniaceae | Simkania | 1 |
| Bacteria | Chloroflexi | Anaerolineae | Anaerolineaeales | Anaerolineaceae | Unclassified | 2 |
| Bacteria | Chloroflexi | Ktedonobacteria | Ktedonobacterales | Unclassified |  | 13.3 |
| Bacteria | Chloroflexi | Ktedonobacteria | Ktedonobacterales | Ktedonobacteraceae | Ktedonobacter | 7.45 |
| Bacteria | Chloroflexi | Ktedonobacteria | Unclassified |  |  | 2.7 |
| Bacteria | Chloroflexi | Unclassified |  |  |  | 23.4 |
| Bacteria | Cyanobacteria/Chloroplast | Chloroplast | Chloroplast | Chloroplast | Chlorophyta | 0.45 |
| Bacteria | Cyanobacteria/Chloroplast | Chloroplast | Chloroplast | Chloroplast | Bacillariophyta | 2.26 |
| Bacteria | Firmicutes | Bacilli | Bacillales | Planococcaceae | Unclassified | 0.2 |
| Bacteria | Firmicutes | Bacilli | Bacillales | Planococcaceae | Kurthia | 0.2 |
| Bacteria | Firmicutes | Bacilli | Lactobacillales | Unclassified |  | 0.2 |
| Bacteria | Firmicutes | Bacilli | Lactobacillales | Leuconostocaceae | Weissella | 0.17 |
| Bacteria | Firmicutes | Bacilli | Lactobacillales | Carnobacteriaceae | Unclassified | 0.1 |
| Bacteria | Firmicutes | Clostridia | Clostridiales | Eubacteriaceae | Acetobacterium | 0.4 |
| Bacteria | Fusobacteria | Fusobacteriia | Fusobacteriales | Fusobacteriaceae | Fusobacterium | 1.2 |
| Bacteria | Fusobacteria | Fusobacteriia | Fusobacteriales | Fusobacteriaceae | Unclassified | 0.6 |
| Bacteria | Gemmatimonadetes | Gemmatimonadetes | Gemmatimonadales | Gemmatimonadaceae | Gemmatimonas | 70 |
| Bacteria | Nitrospirae | Nitrospira | Nitrospirales | Nitrospiraceae | Nitrospira | 25 |
| Bacteria | Planctomycetes | Planctomycetia | Planctomycetales | Planctomycetaceae | Unclassified | 223.7 |
| Bacteria | Planctomycetes | Planctomycetia | Planctomycetales | Planctomycetaceae | Aquisphaera | 0.3 |
| Bacteria | Planctomycetes | Planctomycetia | Planctomycetales | Planctomycetaceae | Gemmata | 33.67 |
| Bacteria | Planctomycetes | Planctomycetia | Unclassified |  |  | 13.3 |
| Bacteria | Planctomycetes | Unclassified |  |  |  | 0.3 |
| Bacteria | Proteobacteria | Alphaproteobacteria | Caulobacterales | Unclassified |  | 0.5 |
| Bacteria | Proteobacteria | Alphaproteobacteria | Caulobacterales | Caulobacteraceae | Unclassified | 1 |
| Bacteria | Proteobacteria | Alphaproteobacteria | Caulobacterales | Caulobacteraceae | Phenylobacterium | 1 |
| Bacteria | Proteobacteria | Alphaproteobacteria | Rhizobiales | Unclassified |  | 2617.1 |
| Bacteria | Proteobacteria | Alphaproteobacteria | Rhizobiales | Bradyrhizobiaceae | Unclassified | 16.7 |
| Bacteria | Proteobacteria | Alphaproteobacteria | Rhizobiales | Bradyrhizobiaceae | Bradyrhizobium | 1.2 |
| Bacteria | Proteobacteria | Alphaproteobacteria | Rhizobiales | Beijerinckiaceae | Unclassified | 36.4 |
| Bacteria | Proteobacteria | Alphaproteobacteria | Rhizobiales | Beijerinckiaceae | Methylocella | 0.5 |
| Bacteria | Proteobacteria | Alphaproteobacteria | Rhizobiales | Rhodobiaceae | Unclassified | 24 |
| Bacteria | Proteobacteria | Alphaproteobacteria | Rhizobiales | Rhodobiaceae | Methyloligella | 43 |
| Bacteria | Proteobacteria | Alphaproteobacteria | Rhizobiales | Xanthobacteraceae | Pseudolabrys | 1 |
| Bacteria | Proteobacteria | Alphaproteobacteria | Rhizobiales | Hyphomicrobiaceae | Unclassified | 27 |
| Bacteria | Proteobacteria | Alphaproteobacteria | Rhizobiales | Hyphomicrobiaceae | Hyphomicrobium | 49 |
| Bacteria | Proteobacteria | Alphaproteobacteria | Rhizobiales | Hyphomicrobiaceae | Rhomicrobium | 3 |
| Bacteria | Proteobacteria | Alphaproteobacteria | Rhizobiales | Hyphomicrobiaceae | Blastochloris | 0.6 |
| Bacteria | Proteobacteria | Alphaproteobacteria | Rhizobiales | Hyphomicrobiaceae | Pedomicrobium | 10.8 |
| Bacteria | Proteobacteria | Alphaproteobacteria | Rhodospirillales | Unclassified |  | 57.6 |
| Bacteria | Proteobacteria | Alphaproteobacteria | Rhodospirillales | Unspecified | Reyranella | 44.7 |
| Bacteria | Proteobacteria | Alphaproteobacteria | Rhodospirillales | Rhodospirillaceae | Unclassified | 123.7 |
| Bacteria | Proteobacteria | Alphaproteobacteria | Rhodospirillales | Rhodospirillaceae | Nitrospirillum | 0.26 |
| Bacteria | Proteobacteria | Alphaproteobacteria | Unclassified |  |  | 105.1 |
| Bacteria | Proteobacteria | Betaproteobacteria | Burkholderiales | Burkholderiaceae | Cupriavidus | 0.4 |
| Bacteria | Proteobacteria | Betaproteobacteria | Burkholderiales | Comamonadaceae | Comamonas | 0.17 |
| Bacteria | Proteobacteria | Betaproteobacteria | Burkholderiales | Oxalobacteraceae | Unclassified | 0.9 |
| Bacteria | Proteobacteria | Betaproteobacteria | Ferrovales | Ferrovaceae | Ferrovum | 3.6 |
| Bacteria | Proteobacteria | Betaproteobacteria | Hydrogenophilales | Hydrogenophilaceae | Thiobacillus | 0.5 |
| Bacteria | Proteobacteria | Betaproteobacteria | Nitrosomonadales | Nitrosomonadaceae | Nitrosomonas | 4 |
| Bacteria | Proteobacteria | Betaproteobacteria | Rhodocyclales | Rhodocyclaceae | Unclassified | 0.3 |
| Bacteria | Proteobacteria | Betaproteobacteria | Rhodocyclales | Rhodocyclaceae | Zoogloea | 0.3 |
| Bacteria | Proteobacteria | Betaproteobacteria | Unclassified |  |  | 187.1 |
| Bacteria | Proteobacteria | Gammaproteobacteria | Alteromonadales | Unclassified |  | 0.15 |
| Bacteria | Proteobacteria | Gammaproteobacteria | Alteromonadales | Pseudoalteromonadaceae | Pseudoalteromonas | 0.2 |
| Bacteria | Proteobacteria | Gammaproteobacteria | Enterobacteriales | Enterobacteriaceae | Unclassified | 3.3 |
| Bacteria | Proteobacteria | Gammaproteobacteria | Legionellales | Coxiellaceae | Aquicella | 1 |
| Bacteria | Proteobacteria | Gammaproteobacteria | Pseudomonadales | Moraxellaceae | Acinetobacter | 0.5 |
| Bacteria | Proteobacteria | Gammaproteobacteria | Pseudomonadales | Pseudomonadaceae | Unclassified | 2.14 |
| Bacteria | Proteobacteria | Gammaproteobacteria | Pseudomonadales | Pseudomonadaceae | Pseudomonas | 0.6 |
| Bacteria | Proteobacteria | Gammaproteobacteria | Xanthomonadales | Unclassified |  | 9.2 |
| Bacteria | Proteobacteria | Gammaproteobacteria | Xanthomonadales | Sinobacteraceae | Unclassified | 20 |
| Bacteria | Proteobacteria | Gammaproteobacteria | Xanthomonadales | Sinobacteraceae | Nevskia | 9.6 |
| Bacteria | Proteobacteria | Gammaproteobacteria | Xanthomonadales | Xanthomonadaceae | Unclassified | 5.6 |
| Bacteria | Proteobacteria | Gammaproteobacteria | Xanthomonadales | Xanthomonadaceae | Stenotrophomonas | 8.25 |
| Bacteria | Proteobacteria | Gammaproteobacteria | Xanthomonadales | Xanthomonadaceae | Lysobacter | 1.6 |
| Bacteria | Proteobacteria | Gammaproteobacteria | Unclassified |  |  | 77.1 |
| Bacteria | Proteobacteria | Deltaproteobacteria | Bdellovibrionales | Bdellovibrionaceae | Vampirovibrio | 5.4 |
| Bacteria | Proteobacteria | Deltaproteobacteria | Myxococcales | Unclassified |  | 77.1 |
| Bacteria | Proteobacteria | Deltaproteobacteria | Myxococcales | Myxococcaceae | Unclassified | 13.1 |
| Bacteria | Proteobacteria | Deltaproteobacteria | Myxococcales | Myxococcaceae | Aggregicoccus | 0.6 |
| Bacteria | Proteobacteria | Deltaproteobacteria | Myxococcales | Poliangaceae | Unclassified | 2.7 |
| Bacteria | Proteobacteria | Deltaproteobacteria | Unclassified |  |  | 10.9 |
| Bacteria | Proteobacteria | Unclassified |  |  |  | 88.74 |
| Bacteria | Unclassified |  |  |  |  | 2149 |
| Bacteria | Verrucomicrobia | Opitutae | Opitutales | Opitutaceae | Unclassified | 1 |
| Bacteria | Verrucomicrobia | Opitutae | Opitutales | Opitutaceae | Opitotus | 4 |
| Bacteria | Verrucomicrobia | Spartobacteria | Spartobacteria_incertae_sedis | Spartobacteria_incertae_sedis | Spartobacteria_genera_incertae_sedis | 4.4 |
| Bacteria | Verrucomicrobia | Spartobacteria | Unclassified |  |  | 0.4 |
| Bacteria | Verrucomicrobia | Unclassified |  |  |  | 0.4 |

^a^ Blank cell refers to the absence of the corresponding taxonomy level

^b^ Number of reads processed using the function of 16S rRNA gene copy number adjustment tool in Ribosomal Database Project (RDP)

**Table S4 Microbial diversity from domain to genus level of the sample S^a^**

| **Domain** | **Phylum** | **Class** | **Order** | **Family** | **Genus** | **# reads^b^** |
| --- | --- | --- | --- | --- | --- | --- |
| Archaea | Crenarchaeota | Thermoprotei | Unclassified |  |  | 6 |
| Archaea | Thaumarchaeota | Nitrososphaerales | Nitrososphaerales | Nitrososphaeraceae | Nitrososphaera | 38 |
| Archaea | Thaumarchaeota | Unclassified |  |  |  | 16 |
| Archaea | Unclassified |  |  |  |  | 29.2 |
| Bacteria | Acidobacteria | Acidobacteria_Gp1 | Acidobacteria_Gp1 | Acidobacteria_Gp1 | Candidatus Koribacter | 27 |
| Bacteria | Acidobacteria | Acidobacteria_Gp1 | Acidobacteria_Gp1 | Acidobacteria_Gp1 | Acidipila | 7 |
| Bacteria | Acidobacteria | Acidobacteria_Gp1 | Acidobacteria_Gp1 | Acidobacteria_Gp1 | Edaphobacter | 2.6 |
| Bacteria | Acidobacteria | Acidobacteria_Gp1 | Acidobacteria_Gp1 | Acidobacteria_Gp1 | Gp1 | 295.6 |
| Bacteria | Acidobacteria | Acidobacteria_Gp1 | Acidobacteria_Gp1 | Acidobacteria_Gp1 | Granulicella | 5 |
| Bacteria | Acidobacteria | Acidobacteria_Gp1 | Unclassified |  |  | 336.3 |
| Bacteria | Acidobacteria | Acidobacteria_Gp2 | Acidobacteria_Gp2 | Acidobacteria_Gp2 | Gp2 | 825 |
| Bacteria | Acidobacteria | Acidobacteria_Gp3 | Acidobacteria_Gp3 | Acidobacteria_Gp3 | Candidatus Solibacter | 4 |
| Bacteria | Acidobacteria | Acidobacteria_Gp3 | Acidobacteria_Gp3 | Acidobacteria_Gp3 | Gp3 | 58 |
| Bacteria | Acidobacteria | Acidobacteria_Gp3 | Acidobacteria_Gp3 | Acidobacteria_Gp3 | Paludibaculum | 1.5 |
| Bacteria | Acidobacteria | Acidobacteria_Gp3 | Unclassified |  |  | 94.5 |
| Bacteria | Acidobacteria | Acidobacteria_Gp4 | Unclassified |  |  | 1.9 |
| Bacteria | Acidobacteria | Acidobacteria_Gp5 | Acidobacteria_Gp5 | Acidobacteria_Gp5 | Gp5 | 1.3 |
| Bacteria | Acidobacteria | Acidobacteria_Gp6 | Acidobacteria_Gp6 | Acidobacteria_Gp6 | Gp6 | 3.8 |
| Bacteria | Acidobacteria | Acidobacteria_Gp7 | Acidobacteria_Gp7 | Acidobacteria_Gp7 | Gp7 | 102.6 |
| Bacteria | Acidobacteria | Acidobacteria_Gp10 | Acidobacteria_Gp10 | Acidobacteria_Gp10 | Gp10 | 3.8 |
| Bacteria | Acidobacteria | Acidobacteria_Gp12 | Acidobacteria_Gp12 | Acidobacteria_Gp12 | Gp12 | 0.6 |
| Bacteria | Acidobacteria | Acidobacteria_Gp13 | Acidobacteria_Gp13 | Acidobacteria_Gp13 | Gp13 | 25.6 |
| Bacteria | Acidobacteria | Acidobacteria_Gp17 | Acidobacteria_Gp17 | Acidobacteria_Gp17 | Gp17 | 1.9 |
| Bacteria | Acidobacteria | Unclassified |  |  |  | 36.5 |
| Bacteria | Actinobacteria | Actinobacteria | Acidimicrobiales | Unclassified |  | 10.5 |
| Bacteria | Actinobacteria | Actinobacteria | Solirubrobacterales | Conexibacteraceae | Conexibacter | 2 |
| Bacteria | Actinobacteria | Actinobacteria | Solirubrobacterales | Unclassified |  | 4 |
| Bacteria | Actinobacteria | Actinobacteria | Actinomycetales | Unclassified |  | 982 |
| Bacteria | Actinobacteria | Actinobacteria | Actinomycetales | Thermomonosporaceae | Actinoallomurus | 0.25 |
| Bacteria | Actinobacteria | Actinobacteria | Actinomycetales | Pseudonocardiaceae | Unclassified | 17.8 |
| Bacteria | Actinobacteria | Actinobacteria | Actinomycetales | Sporichthyaceae | Sporichthya | 33 |
| Bacteria | Actinobacteria | Actinobacteria | Actinomycetales | Micromonosporaceae | Unclassified | 0.6 |
| Bacteria | Actinobacteria | Actinobacteria | Actinomycetales | Mycobacteriaceae | Unclassified | 0.96 |
| Bacteria | Actinobacteria | Actinobacteria | Actinomycetales | Mycobacteriaceae | Mycobacterium | 0.85 |
| Bacteria | Actinobacteria | Actinobacteria | Actinomycetales | Nocardiaceae | Nocardia | 0.3 |
| Bacteria | Actinobacteria | Actinobacteria | Gaiellales | Gaiellaceae | Gaiella | 2 |
| Bacteria | Actinobacteria | Actinobacteria | Unclassified |  |  | 28.5 |
| Bacteria | Actinobacteria | Unclassified |  |  |  | 1 |
| Bacteria | Armatimonadetes | Armatimonadetes_gp4 | Armatimonadetes_gp4 | Armatimonadetes_gp4 | Armatimonadetes_gp4 | 7.4 |
| Bacteria | Armatimonadetes | Chthonomonadetes | Chthonomonadales | Chthonomonadaceae | Chthonomonadas/Armatimonadetes_gp3 | 6 |
| Bacteria | Armatimonadetes | Armatimonadia | Armatimonadales | Armatimonadaceae | Armatimonas/Armatimonadetes_gp1 | 0.46 |
| Bacteria | Bacteroidetes | Bacteroidia | Bacteroidales | Porphyromonadaceae | Unclassified | 0.25 |
| Bacteria | Bacteroidetes | Sphingobacteriia | Sphingobacteriales | Chitinophagaceae | Unclassified | 0.2 |
| Bacteria | Bacteroidetes | Unclassified |  |  |  | 25 |
| Bacteria | Candidate division WPS-2 | Candidate division WPS-2 | Candidate division WPS-2 | Candidate division WPS-2 | WPS-2_genera_incertae_sedis | 0.46 |
| Bacteria | Candidate division WPS-1 | Candidate division WPS-1 | Candidate division WPS-1 | Candidate division WPS-1 | WPS-1_genera_incertae_sedis | 6.48 |
| Bacteria | Chlamydiae | Chlamydiia | Chlamydiales | Unclassified |  | 1.1 |
| Bacteria | Chlamydiae | Chlamydiia | Chlamydiales | Parachlamydiaceae | Unclassified | 1.3 |
| Bacteria | Chloroflexi | Ktedonobacteria | Ktedonobacterales | Unclassified |  | 3.2 |
| Bacteria | Chloroflexi | Ktedonobacteria | Ktedonobacterales | Ktedonobacteraceae | Ktedonobacter | 4.8 |
| Bacteria | Chloroflexi | Ktedonobacteria | Unclassified |  |  | 23.4 |
| Bacteria | Chloroflexi | Unclassified |  |  |  | 9 |
| Bacteria | Cyanobacteria/Chloroplast | Chloroplast | Chloroplast | Chloroplast | Bacillariophyta | 1.4 |
| Bacteria | Cyanobacteria/Chloroplast | Chloroplast | Chloroplast | Chloroplast | Streptophyta | 0.9 |
| Bacteria | Firmicutes | Clostridia | Clostridiales | Unclassified |  | 0.2 |
| Bacteria | Firmicutes | Clostridia | Unclassified |  |  | 0.25 |
| Bacteria | Firmicutes | Clostridia | Thermoanaerobacterales | Thermoanaerobacteraceae | Unclassified | 0.3 |
| Bacteria | Firmicutes | Negativicutes | Selenomonadales | Veillonellaceae | Unclassified | 0.2 |
| Bacteria | Firmicutes | Unclassified |  |  |  | 0.9 |
| Bacteria | Fusobacteria | Fusobacteriia | Fusobacteriales | Fusobacteriaceae | Fusobacterium | 0.2 |
| Bacteria | Fusobacteria | Fusobacteriia | Fusobacteriales | Fusobacteriaceae | Unclassified | 0.15 |
| Bacteria | Gemmatimonadetes | Gemmatimonadetes | Gemmatimonadales | Gemmatimonadaceae | Gemmatimonas | 5 |
| Bacteria | Nitrospirae | Nitrospira | Nitrospirales | Nitrospiraceae | Nitrospira | 9 |
| Bacteria | Planctomycetes | Planctomycetia | Planctomycetales | Planctomycetaceae | Unclassified | 87.3 |
| Bacteria | Planctomycetes | Planctomycetia | Planctomycetales | Planctomycetaceae | Aquisphaera | 2 |
| Bacteria | Planctomycetes | Planctomycetia | Planctomycetales | Planctomycetaceae | Gemmata | 23 |
| Bacteria | Planctomycetes | Planctomycetia | Planctomycetales | Planctomycetaceae | Singulisphaera | 0.88 |
| Bacteria | Planctomycetes | Planctomycetia | Unclassified |  |  | 5 |
| Bacteria | Planctomycetes | Unclassified |  |  |  | 0.3 |
| Bacteria | Proteobacteria | Alphaproteobacteria | Rhodobacterales | Rhodobacteraceae | Unclassified | 0.3 |
| Bacteria | Proteobacteria | Alphaproteobacteria | Rhizobiales | Unclassified |  | 1038.9 |
| Bacteria | Proteobacteria | Alphaproteobacteria | Rhizobiales | Methylocystaceae | Unclassified | 1 |
| Bacteria | Proteobacteria | Alphaproteobacteria | Rhizobiales | Roseiarcaceae | Roseiarcus | 2.6 |
| Bacteria | Proteobacteria | Alphaproteobacteria | Rhizobiales | Bradyrhizobiaceae | Unclassified | 45.6 |
| Bacteria | Proteobacteria | Alphaproteobacteria | Rhizobiales | Bradyrhizobiaceae | Bradyrhizobium | 6.6 |
| Bacteria | Proteobacteria | Alphaproteobacteria | Rhizobiales | Beijerinckiaceae | Unclassified | 16.4 |
| Bacteria | Proteobacteria | Alphaproteobacteria | Rhizobiales | Beijerinckiaceae | Methylocella | 0.5 |
| Bacteria | Proteobacteria | Alphaproteobacteria | Rhizobiales | Rhodobiaceae | Unclassified | 1 |
| Bacteria | Proteobacteria | Alphaproteobacteria | Rhizobiales | Rhodobiaceae | Parvibaculum | 1 |
| Bacteria | Proteobacteria | Alphaproteobacteria | Rhizobiales | Xanthobacteraceae | Pseudolabrys | 0.5 |
| Bacteria | Proteobacteria | Alphaproteobacteria | Rhizobiales | Hyphomicrobiaceae | Unclassified | 13.2 |
| Bacteria | Proteobacteria | Alphaproteobacteria | Rhizobiales | Hyphomicrobiaceae | Hyphomicrobium | 32 |
| Bacteria | Proteobacteria | Alphaproteobacteria | Rhizobiales | Hyphomicrobiaceae | Pedomicrobium | 13.2 |
| Bacteria | Proteobacteria | Alphaproteobacteria | Rhodospirillales | Unclassified |  | 80.5 |
| Bacteria | Proteobacteria | Alphaproteobacteria | Rhodospirillales | Unspecified | Reyranella | 19.5 |
| Bacteria | Proteobacteria | Alphaproteobacteria | Rhodospirillales | Rhodospirillaceae | Unclassified | 53.5 |
| Bacteria | Proteobacteria | Alphaproteobacteria | Rhodospirillales | Rhodospirillaceae | Nitrospirillum | 1 |
| Bacteria | Proteobacteria | Alphaproteobacteria | Rhodospirillales | Acetobacteraceae | Unclassified | 15.1 |
| Bacteria | Proteobacteria | Alphaproteobacteria | Rhodospirillales | Acetobacteraceae | Acidisoma | 1.8 |
| Bacteria | Proteobacteria | Alphaproteobacteria | Alphaproteobacteria_incertae_sedis | Alphaproteobacteria_incertae_sedis | Rhizomicrobium | 8.4 |
| Bacteria | Proteobacteria | Alphaproteobacteria | Unclassified |  |  | 117.7 |
| Bacteria | Proteobacteria | Betaproteobacteria | Burkholderiales | Comamonadaceae | Unclassified | 0.3 |
| Bacteria | Proteobacteria | Betaproteobacteria | Burkholderiales | Alcaligenaceae | Unclassified | 0.8 |
| Bacteria | Proteobacteria | Betaproteobacteria | Hydrogenophilales | Hydrogenophilaceae | Thiobacillus | 0.5 |
| Bacteria | Proteobacteria | Betaproteobacteria | Rhodocyclales | Rhodocyclaceae | Unclassified | 0.6 |
| Bacteria | Proteobacteria | Betaproteobacteria | Unclassified |  |  | 8.2 |
| Bacteria | Proteobacteria | Gammaproteobacteria | Alteromonadales | Alteromonadaceae | Unclassified | 0.3 |
| Bacteria | Proteobacteria | Gammaproteobacteria | Enterobacteriales | Enterobacteriaceae | Unclassified | 0.6 |
| Bacteria | Proteobacteria | Gammaproteobacteria | Enterobacteriales | Enterobacteriaceae | Escherichia/Shigella | 0.1 |
| Bacteria | Proteobacteria | Gammaproteobacteria | Oceanospirillales | Halomonadaceae | Cobetia | 0.2 |
| Bacteria | Proteobacteria | Gammaproteobacteria | Pseudomonadales | Moraxellaceae | Acinetobacter | 0.5 |
| Bacteria | Proteobacteria | Gammaproteobacteria | Pseudomonadales | Moraxellaceae | Unclassified | 0.2 |
| Bacteria | Proteobacteria | Gammaproteobacteria | Pseudomonadales | Pseudomonadaceae | Unclassified | 1.1 |
| Bacteria | Proteobacteria | Gammaproteobacteria | Xanthomonadales | Unclassified |  | 0.8 |
| Bacteria | Proteobacteria | Gammaproteobacteria | Xanthomonadales | Sinobacteraceae | Unclassified | 6.4 |
| Bacteria | Proteobacteria | Gammaproteobacteria | Xanthomonadales | Sinobacteraceae | Nevskia | 2 |
| Bacteria | Proteobacteria | Gammaproteobacteria | Xanthomonadales | Xanthomonadaceae | Unclassified | 6.4 |
| Bacteria | Proteobacteria | Gammaproteobacteria | Xanthomonadales | Xanthomonadaceae | Stenotrophomonas | 3.5 |
| Bacteria | Proteobacteria | Gammaproteobacteria | Xanthomonadales | Xanthomonadaceae | Aspromonas | 0.4 |
| Bacteria | Proteobacteria | Gammaproteobacteria | Xanthomonadales | Xanthomonadaceae | Lysobacter | 0.4 |
| Bacteria | Proteobacteria | Gammaproteobacteria | Unclassified |  |  | 60.2 |
| Bacteria | Proteobacteria | Deltaproteobacteria | Bdellovibrionales | Bdellovibrionaceae | Vampirovibrio | 2.4 |
| Bacteria | Proteobacteria | Deltaproteobacteria | Myxococcales | Unclassified |  | 11 |
| Bacteria | Proteobacteria | Deltaproteobacteria | Myxococcales | Polyangaceae | Unclassified | 0.75 |
| Bacteria | Proteobacteria | Deltaproteobacteria | Unclassified |  |  | 4.1 |
| Bacteria | Proteobacteria | Unclassified |  |  |  | 71.7 |
| Bacteria | Unclassified |  |  |  |  | 1140.9 |
| Bacteria | Verrucomicrobia | Opitutae | Opitutales | Opitutaceae | Opitutus | 19 |
| Bacteria | Verrucomicrobia | Spartobacteria | Spartobacteria_incertae_sedis | Spartobacteria_incertae_sedis | Spartobacteria_genera_incertae_sedis | 20 |
| Bacteria | Verrucomicrobia | Spartobacteria | Unclassified |  |  | 0.9 |
| Bacteria | Verrucomicrobia | Subdivision3 | Subdivision3_incertae_sedis | Subdivision3_incertae_sedis | Subdivision3_genera_incertae_sedis | 4.9 |
| Bacteria | Verrucomicrobia | Unclassified |  |  |  | 1.3 |

^a^ Blank cell refers to the absence of the corresponding taxonomy level

^b^ Number of reads processed using the function of 16S rRNA gene copy number adjustment tool in Ribosomal Database Project (RDP)

**Table S5 Microbial diversity from domain to genus level of the sample WL^a^**

| **Domain** | **Phylum** | **Class** | **Order** | **Family** | **Genus** | **# reads^b^** |
| --- | --- | --- | --- | --- | --- | --- |
| Archaea | Crenarchaeota | Thermoprotei | Unclassified |  |  | 37 |
| Archaea | Thaumarchaeota | Nitrososphaerales | Nitrososphaerales | Nitrososphaeraceae | Nitrososphaera | 7 |
| Archaea | Thaumarchaeota | Unclassified |  |  |  | 6 |
| Archaea | Unclassified |  |  |  |  | 16.8 |
| Bacteria | Acidobacteria | Acidobacteria_Gp1 | Acidobacteria_Gp1 | Acidobacteria_Gp1 | Candidatus Koribacter | 40 |
| Bacteria | Acidobacteria | Acidobacteria_Gp1 | Acidobacteria_Gp1 | Acidobacteria_Gp1 | Acidipila | 1.77 |
| Bacteria | Acidobacteria | Acidobacteria_Gp1 | Acidobacteria_Gp1 | Acidobacteria_Gp1 | Acidobacterium | 11 |
| Bacteria | Acidobacteria | Acidobacteria_Gp1 | Acidobacteria_Gp1 | Acidobacteria_Gp1 | Gp1 | 13.3 |
| Bacteria | Acidobacteria | Acidobacteria_Gp1 | Unclassified |  |  | 52.2 |
| Bacteria | Acidobacteria | Acidobacteria_Gp2 | Acidobacteria_Gp2 | Acidobacteria_Gp2 | Gp2 | 147.4 |
| Bacteria | Acidobacteria | Acidobacteria_Gp3 | Acidobacteria_Gp3 | Acidobacteria_Gp3 | Gp3 | 1.5 |
| Bacteria | Acidobacteria | Acidobacteria_Gp3 | Unclassified |  |  | 5.5 |
| Bacteria | Acidobacteria | Acidobacteria_Gp13 | Acidobacteria_Gp13 | Acidobacteria_Gp13 | Gp13 | 844.2 |
| Bacteria | Acidobacteria | Unclassified |  |  |  | 148.7 |
| Bacteria | Actinobacteria | Actinobacteria | Acidimicrobiales | Unclassified |  | 44 |
| Bacteria | Actinobacteria | Actinobacteria | Acidimicrobiales | Acidimicrobineae_incertae_sedis | Aciditerrimonas | 2 |
| Bacteria | Actinobacteria | Actinobacteria | Solirubrobacterales | Conexibacteraceae | Conexibacter | 1 |
| Bacteria | Actinobacteria | Actinobacteria | Actinomycetales | Unclassified |  | 123.8 |
| Bacteria | Actinobacteria | Actinobacteria | Actinomycetales | Sporichthyaceae | Sporichthya | 1 |
| Bacteria | Actinobacteria | Actinobacteria | Actinomycetales | Mycobacteriaceae | Unclassified | 0.9 |
| Bacteria | Actinobacteria | Actinobacteria | Actinomycetales | Mycobacteriaceae | Mycobacterium | 7.6 |
| Bacteria | Actinobacteria | Actinobacteria | Unclassified |  |  | 16.6 |
| Bacteria | Actinobacteria | Unclassified |  |  |  | 1.01 |
| Bacteria | Armatimonadetes | Armatimonadetes_gp4 | Armatimonadetes_gp4 | Armatimonadetes_gp4 | Armatimonadetes_gp4 | 0.46 |
| Bacteria | Bacteroidetes | Unclassified |  |  |  | 2.3 |
| Bacteria | Candidate division WPS-2 | Candidate division WPS-2 | Candidate division WPS-2 | Candidate division WPS-2 | WPS-2_genera_incertae_sedis | 6.5 |
| Bacteria | Candidate division WPS-1 | Candidate division WPS-1 | Candidate division WPS-1 | Candidate division WPS-1 | WPS-1_genera_incertae_sedis | 2.3 |
| Bacteria | Chloroflexi | Ktedonobacteria | Ktedonobacterales | Unclassified |  | 1.1 |
| Bacteria | Chloroflexi | Ktedonobacteria | Unclassified |  |  | 0.5 |
| Bacteria | Chloroflexi | Unclassified |  |  |  | 3.7 |
| Bacteria | Firmicutes | Clostridia | Clostridiales | Lachnospiraceae | Ruminococcus2 | 1 |
| Bacteria | Firmicutes | Unclassified |  |  |  | 0.9 |
| Bacteria | Planctomycetes | Planctomycetia | Planctomycetales | Planctomycetaceae | Unclassified | 25.3 |
| Bacteria | Planctomycetes | Planctomycetia | Planctomycetales | Planctomycetaceae | Gemmata | 12.3 |
| Bacteria | Planctomycetes | Planctomycetia | Unclassified |  |  | 0.3 |
| Bacteria | Proteobacteria | Alphaproteobacteria | Rhizobiales | Unclassified |  | 1833.8 |
| Bacteria | Proteobacteria | Alphaproteobacteria | Rhizobiales | Methylocystaceae | Unclassified | 1 |
| Bacteria | Proteobacteria | Alphaproteobacteria | Rhizobiales | Roseiarcaceae | Roseiarcus | 0.86 |
| Bacteria | Proteobacteria | Alphaproteobacteria | Rhizobiales | Bradyrhizobiaceae | Nitrobacter | 3 |
| Bacteria | Proteobacteria | Alphaproteobacteria | Rhizobiales | Beijerinckiaceae | Unclassified | 15.6 |
| Bacteria | Proteobacteria | Alphaproteobacteria | Rhizobiales | Beijerinckiaceae | Methylocella | 1 |
| Bacteria | Proteobacteria | Alphaproteobacteria | Rhodospirillales | Unclassified |  | 14.7 |
| Bacteria | Proteobacteria | Alphaproteobacteria | Rhodospirillales | Unspecified | Reyranella | 8.2 |
| Bacteria | Proteobacteria | Alphaproteobacteria | Rhodospirillales | Rhodospirillaceae | Unclassified | 9.7 |
| Bacteria | Proteobacteria | Alphaproteobacteria | Rhodospirillales | Acetobacteraceae | Unclassified | 61.8 |
| Bacteria | Proteobacteria | Alphaproteobacteria | Rhodospirillales | Acetobacteraceae | Acidisoma | 35.3 |
| Bacteria | Proteobacteria | Alphaproteobacteria | Alphaproteobacteria_incertae_sedis | Alphaproteobacteria_incertae_sedis | Rhizomicrobium | 7.4 |
| Bacteria | Proteobacteria | Alphaproteobacteria | Unclassified |  |  | 102.8 |
| Bacteria | Proteobacteria | Betaproteobacteria | Burkholderiales | Unclassified |  | 1.1 |
| Bacteria | Proteobacteria | Betaproteobacteria | Hydrogenophilales | Hydrogenophilaceae | Thiobacillus | 0.5 |
| Bacteria | Proteobacteria | Betaproteobacteria | Rhodocyclales | Rhodocyclaceae | Unclassified | 0.3 |
| Bacteria | Proteobacteria | Gammaproteobacteria | Enterobacteriales | Enterobacteriaceae | Unclassified | 0.15 |
| Bacteria | Proteobacteria | Gammaproteobacteria | Legionellales | Coxiellaceae | Aquicella | 53 |
| Bacteria | Proteobacteria | Gammaproteobacteria | Unclassified |  |  | 17.2 |
| Bacteria | Proteobacteria | Deltaproteobacteria | Bdellovibrionales | Bdellovibrionaceae | Vampirovibrio | 1.2 |
| Bacteria | Proteobacteria | Deltaproteobacteria | Desulphovibrionales | Desulphovibrionaceae | Unclassified | 0.36 |
| Bacteria | Proteobacteria | Deltaproteobacteria | Myxococcales | Unclassified |  | 1 |
| Bacteria | Proteobacteria | Unclassified |  |  |  | 25.6 |
| Bacteria | Unclassified |  |  |  |  | 897.9 |
| Bacteria | Verrucomicrobia | Opitutae | Opitutales | Opitutaceae | Opitotus | 4 |

^a^ Blank cell refers to the absence of the corresponding taxonomy level

^b^ Number of reads processed using the function of 16S rRNA gene copy number adjustment tool in Ribosomal Database Project (RDP)

**Table S6 Microbial diversity from domain to genus level of the sample F^a^**

| **Domain** | **Phylum** | **Class** | **Order** | **Family** | **Genus** | **# reads^b^** |
| --- | --- | --- | --- | --- | --- | --- |
| Archaea | Crenarchaeota | Thermoprotei | Unclassified |  |  | 12 |
| Archaea | Thaumarchaeota | Nitrososphaerales | Nitrososphaerales | Nitrososphaeraceae | Nitrososphaera | 1 |
| Archaea | Unclassified |  |  |  |  | 5.3 |
| Bacteria | Acidobacteria | Acidobacteria_Gp1 | Acidobacteria_Gp1 | Acidobacteria_Gp1 | Acidipila | 1.7 |
| Bacteria | Acidobacteria | Acidobacteria_Gp1 | Acidobacteria_Gp1 | Acidobacteria_Gp1 | Acidobacterium | 14 |
| Bacteria | Acidobacteria | Acidobacteria_Gp1 | Acidobacteria_Gp1 | Acidobacteria_Gp1 | Terriglobus | 2 |
| Bacteria | Acidobacteria | Acidobacteria_Gp1 | Unclassified |  |  | 57.5 |
| Bacteria | Acidobacteria | Acidobacteria_Gp2 | Acidobacteria_Gp2 | Acidobacteria_Gp2 | Gp2 | 22.4 |
| Bacteria | Acidobacteria | Acidobacteria_Gp13 | Acidobacteria_Gp13 | Acidobacteria_Gp13 | Gp13 | 41 |
| Bacteria | Acidobacteria | Acidobacteria_Gp14 | Acidobacteria_Gp14 | Acidobacteria_Gp14 | Gp14 | 3.8 |
| Bacteria | Acidobacteria | Unclassified |  |  |  | 10.2 |
| Bacteria | Actinobacteria | Actinobacteria | Acidimicrobiales | Unclassified |  | 100 |
| Bacteria | Actinobacteria | Actinobacteria | Acidimicrobiales | Acidimicrobineae_incertae_sedis | Aciditerrimonas | 5.5 |
| Bacteria | Actinobacteria | Actinobacteria | Solirubrobacterales | Conexibacteraceae | Conexibacter | 1 |
| Bacteria | Actinobacteria | Actinobacteria | Solirubrobacterales | Unclassified |  | 4 |
| Bacteria | Actinobacteria | Actinobacteria | Actinomycetales | Unclassified |  | 1575.8 |
| Bacteria | Actinobacteria | Actinobacteria | Actinomycetales | Micromonosporaceae | Unclassified | 13.4 |
| Bacteria | Actinobacteria | Actinobacteria | Actinomycetales | Pseudonocardiaceae | Unclassified | 0.8 |
| Bacteria | Actinobacteria | Actinobacteria | Actinomycetales | Sporichthyaceae | Sporichthya | 0.5 |
| Bacteria | Actinobacteria | Actinobacteria | Actinomycetales | Mycobacteriaceae | Unclassified | 5.3 |
| Bacteria | Actinobacteria | Actinobacteria | Actinomycetales | Mycobacteriaceae | Mycobacterium | 16.1 |
| Bacteria | Actinobacteria | Actinobacteria | Unclassified |  |  | 81.5 |
| Bacteria | Actinobacteria | Unclassified |  |  |  | 3 |
| Bacteria | Bacteroidetes | Unclassified |  |  |  | 0.7 |
| Bacteria | Candidate division WPS-2 | Candidate division WPS-2 | Candidate division WPS-2 | Candidate division WPS-2 | WPS-2_genera_incertae_sedis | 19 |
| Bacteria | Firmicutes | Unclassified |  |  |  | 0.2 |
| Bacteria | Nitrospirae | Nitrospira | Nitrospirales | Nitrospiraceae | Nitrospira | 6 |
| Bacteria | Planctomycetes | Planctomycetia | Planctomycetales | Planctomycetaceae | Unclassified | 10.7 |
| Bacteria | Planctomycetes | Planctomycetia | Planctomycetales | Planctomycetaceae | Gemmata | 6 |
| Bacteria | Proteobacteria | Alphaproteobacteria | Caulobacterales | Caulobacteraceae | Phenylobacterium | 4 |
| Bacteria | Proteobacteria | Alphaproteobacteria | Rhizobiales | Unclassified |  | 72.6 |
| Bacteria | Proteobacteria | Alphaproteobacteria | Rhizobiales | Bradyrhizobiaceae | Unclassified | 1.5 |
| Bacteria | Proteobacteria | Alphaproteobacteria | Rhizobiales | Bradyrhizobiaceae | Nitrobacter | 3 |
| Bacteria | Proteobacteria | Alphaproteobacteria | Rhizobiales | Beijerinckiaceae | Unclassified | 10 |
|  |  |  |  |  | Methylovirgula | 2.4 |
| Bacteria | Proteobacteria | Alphaproteobacteria | Rhodospirillales | Unclassified |  | 11.3 |
| Bacteria | Proteobacteria | Alphaproteobacteria | Rhodospirillales | Rhodospirillaceae | Unclassified | 0.5 |
|  |  |  |  | SAR11 | Candidatus Pelagibacter | 1 |
| Bacteria | Proteobacteria | Alphaproteobacteria | Rhodospirillales | Acetobacteraceae | Unclassified | 104.2 |
| Bacteria | Proteobacteria | Alphaproteobacteria | Rhodospirillales | Acetobacteraceae | Acidisoma | 0.5 |
| Bacteria | Proteobacteria | Alphaproteobacteria | Unclassified |  |  | 23.7 |
|  |  |  |  |  | Janthinobacterium | 1 |
| Bacteria | Proteobacteria | Betaproteobacteria | Burkholderiales | Unclassified |  | 0.4 |
| Bacteria | Proteobacteria | Betaproteobacteria | Nitrosomonadales | Nitrosomonadaceae | Nitrosomonas | 1 |
| Bacteria | Proteobacteria | Betaproteobacteria | Unclassified |  |  | 1.4 |
| Bacteria | Proteobacteria | Gammaproteobacteria | Enterobacteriales | Enterobacteriaceae | Unclassified | 0.3 |
| Bacteria | Proteobacteria | Gammaproteobacteria | Legionellales | Coxiellaceae | Aquicella | 6 |
| Bacteria | Proteobacteria | Gammaproteobacteria | Xanthomonadales | Xanthomonadaceae | Unclassified | 1.6 |
| Bacteria | Proteobacteria | Gammaproteobacteria | Xanthomonadales | Xanthomonadaceae | Fulvimonas | 0.4 |
| Bacteria | Proteobacteria | Gammaproteobacteria | Unclassified |  |  | 24.2 |
| Bacteria | Proteobacteria | Unclassified |  |  |  | 13 |
| Bacteria | Unclassified |  |  |  |  | 811.6 |

^a^ Blank cell refers to the absence of the corresponding taxonomy level

^b^ Number of reads processed using the function of 16S rRNA gene copy number adjustment tool in Ribosomal Database Project (RDP)

**Table S7 Microbial diversity from domain to genus level of the sample WB^a^**

| **Domain** | **Phylum** | **Class** | **Order** | **Family** | **Genus** | **# reads^b^** |
| --- | --- | --- | --- | --- | --- | --- |
| Archaea | Crenarchaeota | Thermoprotei | Unclassified |  |  | 40 |
| Archaea | Thaumarchaeota | Nitrososphaerales | Nitrososphaerales | Nitrososphaeraceae | Nitrososphaera | 3 |
| Archaea | Thaumarchaeota | Unclassified |  |  |  | 5 |
| Archaea | Unclassified |  |  |  |  | 10.6 |
| Bacteria | Acidobacteria | Acidobacteria_Gp1 | Acidobacteria_Gp1 | Acidobacteria_Gp1 | Candidatus Koribacter | 1 |
| Bacteria | Acidobacteria | Acidobacteria_Gp1 | Acidobacteria_Gp1 | Acidobacteria_Gp1 | Gp1 | 11.5 |
| Bacteria | Acidobacteria | Acidobacteria_Gp1 | Unclassified |  |  | 9.7 |
| Bacteria | Acidobacteria | Acidobacteria_Gp2 | Acidobacteria_Gp2 | Acidobacteria_Gp2 | Gp2 | 107 |
| Bacteria | Acidobacteria | Acidobacteria_Gp3 | Acidobacteria_Gp3 | Acidobacteria_Gp3 | Gp3 | 4 |
| Bacteria | Acidobacteria | Acidobacteria_Gp3 | Unclassified |  |  | 1 |
| Bacteria | Acidobacteria | Acidobacteria_Gp6 | Acidobacteria_Gp6 | Acidobacteria_Gp6 | Gp6 | 4.5 |
| Bacteria | Acidobacteria | Acidobacteria_Gp13 | Acidobacteria_Gp13 | Acidobacteria_Gp13 | Gp13 | 54.5 |
| Bacteria | Acidobacteria | Acidobacteria_Gp17 | Acidobacteria_Gp17 | Acidobacteria_Gp17 | Gp17 | 1.9 |
| Bacteria | Acidobacteria | Unclassified |  |  |  | 14.7 |
| Bacteria | Actinobacteria | Actinobacteria | Acidimicrobiales | Unclassified |  | 2.5 |
| Bacteria | Actinobacteria | Actinobacteria | Actinomycetales | Unclassified |  | 79.5 |
| Bacteria | Actinobacteria | Actinobacteria | Actinomycetales | Pseudonocardiaceae | Unclassified | 19.4 |
| Bacteria | Actinobacteria | Actinobacteria | Actinomycetales | Sporichthyaceae | Sporichthya | 0.5 |
| Bacteria | Actinobacteria | Actinobacteria | Actinomycetales | Micromonosporaceae | Unclassified | 0.6 |
| Bacteria | Actinobacteria | Actinobacteria | Unclassified |  |  | 1 |
| Bacteria | Armatimonadetes | Armatimonadetes_gp4 | Armatimonadetes_gp4 | Armatimonadetes_gp4 | Armatimonadetes_gp4 | 0.5 |
| Bacteria | Armatimonadetes | Armatimonadetes_gp5 | Armatimonadetes_gp5 | Armatimonadetes_gp5 | Armatimonadetes_gp5 | 0.9 |
| Bacteria | Armatimonadetes | Chthonomonadetes | Chthonomonadales | Chthonomonadaceae | Chthonomonadas/Armatimonadetes_gp3 | 3.2 |
| Bacteria | Candidate division WPS-2 | Candidate division WPS-2 | Candidate division WPS-2 | Candidate division WPS-2 | WPS-2_genera_incertae_sedis | 2.8 |
| Bacteria | Chloroflexi | Ktedonobacteria | Ktedonobacterales | Ktedonobacteraceae | Ktedonobacter | 0.5 |
| Bacteria | Chloroflexi | Unclassified |  |  |  | 2.7 |
| Bacteria | Gemmatimonadetes | Gemmatimonadetes | Gemmatimonadales | Gemmatimonadaceae | Gemmatimonas | 4 |
| Bacteria | Planctomycetes | Planctomycetia | Planctomycetales | Planctomycetaceae | Unclassified | 18.7 |
| Bacteria | Planctomycetes | Planctomycetia | Planctomycetales | Planctomycetaceae | Schlesneria | 0.3 |
| Bacteria | Planctomycetes | Planctomycetia | Planctomycetales | Planctomycetaceae | Aquisphaera | 1.3 |
| Bacteria | Planctomycetes | Planctomycetia | Planctomycetales | Planctomycetaceae | Gemmata | 6.7 |
| Bacteria | Planctomycetes | Planctomycetia | Unclassified |  |  | 1 |
| Bacteria | Planctomycetes | Unclassified |  |  |  | 0.3 |
| Bacteria | Proteobacteria | Alphaproteobacteria | Caulobacterales | Caulobacteraceae | Unclassified | 0.5 |
| Bacteria | Proteobacteria | Alphaproteobacteria | Rhizobiales | Unclassified |  | 374.8 |
| Bacteria | Proteobacteria | Alphaproteobacteria | Rhizobiales | Roseiarcaceae | Roseiarcus | 2.6 |
| Bacteria | Proteobacteria | Alphaproteobacteria | Rhizobiales | Bradyrhizobiaceae | Unclassified | 0.7 |
| Bacteria | Proteobacteria | Alphaproteobacteria | Rhizobiales | Bradyrhizobiaceae | Bradyrhizobium | 0.6 |
| Bacteria | Proteobacteria | Alphaproteobacteria | Rhizobiales | Beijerinckiaceae | Unclassified | 4.8 |
| Bacteria | Proteobacteria | Alphaproteobacteria | Rhizobiales | Beijerinckiaceae | Methylocella | 1 |
| Bacteria | Proteobacteria | Alphaproteobacteria | Rhizobiales | Rhizobiaceae | Rhizobium | 3.6 |
| Bacteria | Proteobacteria | Alphaproteobacteria | Rhizobiales | Hyphomicrobiaceae | Unclassified | 4.8 |
| Bacteria | Proteobacteria | Alphaproteobacteria | Rhizobiales | Hyphomicrobiaceae | Hyphomicrobium | 4 |
| Bacteria | Proteobacteria | Alphaproteobacteria | Rhodospirillales | Unclassified |  | 22.4 |
| Bacteria | Proteobacteria | Alphaproteobacteria | Rhodospirillales | Unspecified | Reyranella | 20 |
| Bacteria | Proteobacteria | Alphaproteobacteria | Rhodospirillales | Rhodospirillaceae | Unclassified | 23.2 |
| Bacteria | Proteobacteria | Alphaproteobacteria | Rhodospirillales | Acetobacteraceae | Unclassified | 14.6 |
| Bacteria | Proteobacteria | Alphaproteobacteria | Rhodospirillales | Acetobacteraceae | Acidisoma | 2.9 |
| Bacteria | Proteobacteria | Alphaproteobacteria | Alphaproteobacteria_incertae_sedis | Alphaproteobacteria_incertae_sedis | Rhizomicrobium | 5.6 |
| Bacteria | Proteobacteria | Alphaproteobacteria | Unclassified |  |  | 28.4 |
| Bacteria | Proteobacteria | Betaproteobacteria | Burkholderiales | Burkholderiaceae | Cupriavidus | 3.8 |
| Bacteria | Proteobacteria | Betaproteobacteria | Burkholderiales |  | Unclassified | 0.27 |
| Bacteria | Proteobacteria | Betaproteobacteria | Burkholderiales |  | Delftia | 0.4 |
| Bacteria | Proteobacteria | Betaproteobacteria | Burkholderiales | Comamonadaceae | Unclassified | 6.5 |
| Bacteria | Proteobacteria | Betaproteobacteria | Burkholderiales | Oxalobacteraceae | Unclassified | 36 |
| Bacteria | Proteobacteria | Betaproteobacteria | Burkholderiales |  | Janthinobacterium | 2013 |
| Bacteria | Proteobacteria | Betaproteobacteria | Burkholderiales | Alcaligenaceae | Unclassified | 0.4 |
| Bacteria | Proteobacteria | Betaproteobacteria | Burkholderiales | Unclassified |  | 11.3 |
| Bacteria | Proteobacteria | Betaproteobacteria | Unclassified |  |  | 30.5 |
| Bacteria | Proteobacteria | Gammaproteobacteria | Alteromonadales | Unclassified |  | 40.5 |
| Bacteria | Proteobacteria | Gammaproteobacteria | Enterobacteriales | Enterobacteriaceae | Unclassified | 425.5 |
| Bacteria | Proteobacteria | Gammaproteobacteria | Enterobacteriales | Enterobacteriaceae | Enterobacter | 0.1 |
| Bacteria | Proteobacteria | Gammaproteobacteria | Enterobacteriales | Enterobacteriaceae | Cedecea | 1.2 |
| Bacteria | Proteobacteria | Gammaproteobacteria | Enterobacteriales | Enterobacteriaceae | Salmonella | 0.1 |
| Bacteria | Proteobacteria | Gammaproteobacteria | Pseudomonadales | Moraxellaceae | Acinetobacter | 0.2 |
| Bacteria | Proteobacteria | Gammaproteobacteria | Pseudomonadales | Pseudomonadaceae | Unclassified | 13 |
| Bacteria | Proteobacteria | Gammaproteobacteria | Pseudomonadales | Pseudomonadaceae | Pseudomonas | 19.76 |
| Bacteria | Proteobacteria | Gammaproteobacteria | Pseudomonadales | Unclassified |  | 0.2 |
| Bacteria | Proteobacteria | Gammaproteobacteria | Xanthomonadales | Sinobacteraceae | Unclassified | 0.4 |
| Bacteria | Proteobacteria | Gammaproteobacteria | Xanthomonadales | Xanthomonadaceae | Fulvimonas | 1.2 |
| Bacteria | Proteobacteria | Deltaproteobacteria | Bdellovibrionales | Bdellovibrionaceae | Vampirovibrio | 5.4 |
| Bacteria | Proteobacteria | Deltaproteobacteria | Myxococcales | Unclassified |  | 2.3 |
| Bacteria | Proteobacteria | Deltaproteobacteria | Myxococcales | Polyangaceae | Unclassified | 0.25 |
| Bacteria | Proteobacteria | Deltaproteobacteria | Unclassified |  |  | 3.2 |
| Bacteria | Proteobacteria | Unclassified |  |  |  | 46.1 |
| Bacteria | Unclassified |  |  |  |  | 316.9 |

^a^ Blank cell refers to the absence of the corresponding taxonomy level

^b^ Number of reads processed using the function of 16S rRNA gene copy number adjustment tool in Ribosomal Database Project (RDP)
